# Genetic Dissection of the General Combining Ability of Yield-Related Traits in Maize

**DOI:** 10.3389/fpls.2020.00788

**Published:** 2020-07-22

**Authors:** Xin Lu, Zhiqiang Zhou, Zhaohui Yuan, Chaoshu Zhang, Zhuanfang Hao, Zhenhua Wang, Mingshun Li, Degui Zhang, Hongjun Yong, Jienan Han, Xinhai Li, Jianfeng Weng

**Affiliations:** ^1^College of Agriculture, Northeast Agricultural University, Harbin, China; ^2^Institute of Crop Science, Chinese Academy of Agricultural Sciences, Beijing, China

**Keywords:** maize, yield-related traits, NCII mating design, combining ability, QTL

## Abstract

Maize yield components including row number, kernel number per row, kernel thickness, kernel width, kernel length, 100-kernel weight, and volume weight affect grain yield directly. Previous studies mainly focused on dissecting the genetic basis of *per se* performances for yield-related traits, but the genetic basis of general combining ability (GCA) for these traits is still unclear. In the present study, 328 RILs were crossed as males to two testers according to the NCII mating design, resulting in a hybrid panel composed of 656 hybrids. Both the hybrids and parental lines were evaluated in four environments in 2015 and 2016. Correlation analysis showed the performances of GCA effects were significantly correlated to the *per se* performances of RILs for all yield-related traits (0.17 ≤ *r* ≤ 0.64, *P* > 0.01). Only 17 of 95 QTL could be detected for both *per se* performances of RILs and GCA effects for eight yield-related traits. The QTL *qKN7-1* and *qHKW1-3*, which could explain more than 10% of the variation in the GCA effects of KN and HKW, were also detected for *per se* performances for the traits. The pleiotropic loci *qRN3-1* and *qRN6*, which together explained 14.92% of the observed variation in GCA effects for RN, were associated with the GCA effects of KW and HKW, but not with *per se* performances for these traits. In contrast, *Incw1*, which was related to seed weight in maize, was mapped to the region surrounding MK2567 at the *qHKW5-2* locus, but no GCA effect was detected. The QTL identified in present study for *per se* performances and corresponding GCA effects for yield-related traits might be useful for maize hybrid breeding.

## Introduction

Maize (*Zea mays* L.), one of the most extensively grown crops worldwide, is important for both animal feed and as a bioenergy feedstock. Crop breeders and farmers face the daunting task of meeting increasing demand for food and feed. The improvement of maize yield depends mainly on the exploitation of heterosis in the F_1_ resulting from the hybridization of genetically diverse parents. However, the yield of hybrids cannot be predicted by the performance of their parents *per se* (Hallauer, 1990), but depends largely on their combining ability, or the potential to generate progenies exhibiting high heterosis. Thus, understanding the genetic basis of their combining ability is essential for utilizing heterosis and selecting elite inbred lines in hybrid breeding.

Combining ability can be divided into general combining ability (GCA) and specific combining ability (SCA). The genetic variances of GCA and SCA were first estimated using a diallel cross-mating design (Griffing, 1956). GCA is estimated by the average performance of parents, while SCA is measured as the deviations of hybrid performance from that predicted from the GCA of the parents (Sprague and Tatum, 1942; Xu, 2010). GCA is primarily the result of additive allelic effects of high-heritability traits, and SCA mainly results from non-additive effects including dominance and epistasis (Reif et al., 2007). Due to their low heritability and interactions with the environment, the non-additive effects usually are difficult to resolve in progeny (Lu, 1999). Therefore, during the crop breeding process, the contribution of GCA to test-cross hybrid values and inbred line development is greater than that of SCA (Schrag et al., 2006; Fischer et al., 2010; Grieder et al., 2012).

Combining ability estimates have been used to anticipate improvement due to hybridization and selection in maize breeding (Khalil et al., 2010; Issa et al., 2018), but further exploration will be required to dissect the genetic basis of combining ability (Gichuru et al., 2017). With the development of molecular markers (e.g., AFLP, RFLP, SSR), the genetic basis of combining ability effects was estimated by QTL mapping (Liu et al., 2004; Basbag et al., 2007; Shukla and Pandey, 2010; Giraud et al., 2017; Zhou Q. et al., 2017; Seye et al., 2019). In maize, 23 QTL related to the GCA effects of 10 yield-related traits were identified using a linkage map constructed from 146 pairs of SSR primers using a double haploid (DH) population (Gu, 2007). A total of 21 loci for SCA and 56 significant loci for GCA have been identified for five yield-related traits in multiple environments using a set of testcrosses with introgression lines (ILs) (Qi et al., 2013). *Ghd7* and *OsPRR37* were confirmed to affect the GCA effects of three yield-related traits in rice by linkage analysis (Liu et al., 2015). These studies indicated that using molecular markers to construct linkage maps was an effective way to dissect the genetic basis of combining ability. However, the resolution of those genetic maps based on traditional molecular markers was relatively low. Fine-scale mapping was more time-consuming, identifying QTL with minimal effects was more challenging (Holland, 2007). Next-generation sequencing technologies for constructing high-density genetic maps for large QTL mapping population are more efficient and improve mapping accuracy (Chen et al., 2014; Zhou et al., 2016). For example, *qPH5-1* and *qPH10*, which are located on chromosomes 5 and 10, respectively, were validated for maize plant height-related traits using a high-density genetic linkage map containing 4602 bin markers developed using genotyping by sequencing (GBS). The genotype at the *qPH10* locus was only associated with GCA effects, while that at *qPH5-1* was associated with both *per se* performances and corresponding GCA effects for the traits (Zhou et al., 2018). A recent genome-wide association study (GWAS) identified 34 significant associations between GCA and SCA, and agronomic traits in rice, and suggested the accumulation of desirable *Ghd8*, *GS3*, and *qSSR4* alleles in parental lines with high GCA (Chen et al., 2019). Chromosome segment substitution lines (CSSLs) were elite genetic resources for identifying naturally occurring favorable alleles. A total of 40 significant GCA loci were identified for 14 grain and stover yield-related traits in millet using testcross hybrid populations of 85 CSSLs (Basava et al., 2019). Differing from the trait values obtained by observation and measurement, GCA effects were statistical values, and the feasibility of analyzing the genetic basis of GCA effects by molecular markers has been proved theoretically and practically.

Grain yield is a complex quantitative trait that cannot be improved by directly selecting individual plants with excellent performance. This is especially true for heterozygous maize, for which yield gains largely depend on the utilization of heterosis (Messmer et al., 2009). Previous studies have identified highly significant genetic correlations between grain yield and yield components, including kernel number per ear, kernel weight, and volume weight (Ahmed et al., 1992; Zhang et al., 2007; Li C.H. et al., 2013; Liu et al., 2014; Liu C.L. et al., 2016; Liu et al., 2015). The factors, kernel number per row (KN) and kernel row number (RN), are the two components of kernel number per ear. Kernel weight is mainly determined by kernel thickness (KT), kernel width (KW), and kernel length (KL). Comparing with grain yield, these yield components exhibit higher heritability (Messmer et al., 2009; Li C.H. et al., 2013; Raihan et al., 2016). Although numerous QTL have been identified to associate with *per se* performances for yield-related traits in maize, few studies concentrate on the genetic basis of the GCA for yield-related traits (Zhang et al., 2017; Zhou Q. et al., 2017; Lan et al., 2018). Therefore, it is essential to study the genetic basis of yield components and their combining abilities, especially GCA, for the improvement of grain yield.

In the present study, a NCII mating design was used to analysis the GCA effects of eight yield-related traits in 328 RILs and two test cross-populations by linkage analysis across four environments. The objectives of this study were to (a) analyze the correlation between *per se* performances and GCA effects for yield-related traits; (b) identify stable QTL for GCA of these traits; (c) compare the genetic basis of *per se* performances and GCA effects for these traits and discuss the utility of these QTL for maize breeding.

## Materials and Methods

### Materials

A set of 365 RILs and two testers were analyzed in the present study. These RILs were derived from a cross between Ye478 (as female) and Qi319 (as male). Ye478 and Qi319, two elite inbred lines, were selected from the PA (Partner A) and PB (Partner B) heterotic groups, respectively. The two testers were Chang7-2 and Mo17, which belong to the SPT and Lancaster heterotic groups, respectively. Chang7-2, an inbred line derived from Huangzao4, was extensively used in the Yellow and Huai River maize-growing zone of China. Mo17, an elite inbred line derived from Lancaster Sure Crop, has been used widely in commercial maize breeding. Each RIL was crossed as male to the two testers according to the NCII mating design. A hybrid panel composed of 656 hybrids was thus obtained by 328 RILs that were successfully crossed with both Chang7-2 and Mo17, as sufficient seed was not produced from 74 crosses. The Chang7-2 testcross population and Mo17 testcross population were defined as TC and TM, respectively. The four F_1_ hybrids that were produced by crossing the Ye478 and Qi319 to the two testers were used as controls.

### Field Experiments

Phenotypic performance was evaluated in 2015 and 2016 at Shijiazhuang (37.27°N, 113.30°E) and Xinxiang (35.19°N, 113.53°E) in China. Both of the locations for evaluating the phenotypic performance of the parental lines and hybrids were located in the summer maize-growing regions of China. All of the experimental materials were arranged in a randomized incomplete block design with two-row plots and two replicates at each location. In each row, 17 plants were planted with 25 cm of space between each plant, 60 cm of space between rows, and a final density of 60,000 plants/ha. RILs and testcrosses were planted separately to avoid the competitive effects. Fields were managed according to the standard agronomic practices for maize.

### Evaluation of Phenotypes

At harvest stage, the middle 10 open-pollinated ears in the central row of each plot were sampled for phenotypic evaluation using standard procedures after air-drying. Eight agronomic traits were measured in this study, including kernel thickness (KT, mm), kernel width (KW, mm), kernel length (KL, mm), 100-kernel weight (HKW, g), row number (RN), kernel number per row (KN), volume weight (VW), and yield per plot (YP, g). An electronic digital caliper with a precision of 0.1 mm was used to measure the former three traits, and these traits were scored by randomly selecting 10 kernels from the middle of each ear. HKW was measured as three repeated measurements of 100 kernels mixed from 10 ears per plot. RN, KN, VW, and YP were determined from 10 randomly selected ears. The average measured value of three replications for each trait in each environment represents the performance for each plot.

### Statistical Analysis of Phenotypic Data

The “‘*lme*” function in the R package “*lme4*” was applied to correct the raw phenotypic data using best linear unbiased estimation (BLUE) using the formula: Pheno ∼1 + Line + (1| Year) + (1| Loc) + (1| Line: Year) + (1| Line: Loc) + (1| Rep), where Pheno represents trait data; Line represents the phenotypic data of inbred lines or hybrids; Year represents the planting year; Loc represents the planting location; and Rep represents the replications in each environment. Line is considered a fixed effect, while the other factors are considered random effects: indicates an interaction between factors, and | separates the model matrix and grouping factors. Estimates of phenotypic distributions, correlations, and part of these QTL analyses were based on BLUE.

The broad-sense heritability (*H*^2^) for each of the traits analyzed in the RILs across multiple environments was estimated according to Knapp (Knapp et al., 1985) using the modified formula: *H*^2^ = σ*^2^_*G*_*/(σ*^2^_*G*_* + σ*^2^_*GL*_/L* + σ*^2^_*GY*_/Y* + σ*^2^_*GLY*_*/*L* × *Y* + σ*^2^_*E*_*/*L* × *Y* × *R*), where σ*^2^_*G*_* is the genotypic variance; σ*^2^_*GL*_*,σ*^2^_*GY*_* and σ*^2^_*GLY*_* are estimates of genotype × location interaction variance, genotype × year interaction variance, and genotype × location × year interaction variance; σ*^2^_*E*_* is the error variance; *L* is the number of location; *Y* is the number of year; and *R* refers to the number of replications per location, respectively. All of these variances were estimated using the “*ASReml*” R package. The genetic variance effects of GCA and SCA of both testcross populations in four environments were evaluated using a joint linear mixed model (Butler et al., 2007), as follows:

$$\begin{array}{ll} Y_{ijklm} & =\text{μ}+L_{i}+Y_{m}+B_{j(im)}+GCA_{k}+GCA_{l}\\ & +SCA_{kl}+L\times GCA_{ik}+L\times GCA_{il}\\ & +L\times SCA_{ikl}+Y\times GCA_{ik}+Y\times GCA_{il}\\ & +Y\times SCA_{ikl}+L\times Y\times GCA_{ik}\\ & +L\times Y\times GCA_{il}+L\times Y\times SCA_{ikl}+Ei_{jklm}, \end{array}$$

where *Y*_*ijklm*_ indicates the phenotypic value of the hybrid derived from the *k*th female and the *l*th male evaluated in the *j*th block and the *i*th location of the *m*th year; μ refers to the overall mean, *L*_*i*_ is the *i*th location effect; *Y*_*m*_ is the *m*th year effect; and *B*_*j(im)*_ indicates the *j*th block within the *i*th location and *m*th year. *GCA*_*k*_ and *GCA*_*l*_ are the effects of *k*th female and the *l*th male, respectively; *SCA*_*kl*_ is the effect of the *k*th and the *l*th parents; *L* × *GCA*_*ik*_, *L* × *GCA*_*il*_, *L* × *SCA*_*ikl*_ indicates the location interaction effect by *GCA*_*ik*_, *GCA*_*il*_, and *SCA*_*ikl*_, respectively; *Y* × *GCA*_*ik*_, *Y* × *GCA*_*il*_, *Y* × *SCA*_*ikl*_ indicates the year interaction effect by *GCA*_*ik*_, *GCA*_*il*_, and *SCA*_*ikl*_, respectively; *L* × *Y* × *GCA*_*ik*_, *L* × *Y* × *GCA*_*il*_, *L* × *Y* × *SCA*_*ikl*_ indicates the location and the year interaction effect by *GCA*_*ik*_, *GCA*_*il*_, and *SCA*_*ikl*_, respectively; and *E*_*ijklm*_ is random error. *L*_*i*_ and the remaining factors are considered random effects.

### Linkage Mapping and QTL Detection

A high-density linkage map was constructed for a population of 365 F_11_ RILs using genotyping by sequencing (GBS) technology on an Illumina 2500 platform and the raw sequence reads of these lines are public on NCBI (Accession: PRJNA627044)^1^. We developed a total of 88,268 SNP markers from SNP sites that were heterozygous between parental lines and used them to genotype this population. Finally, 4602 high-quality bin markers were obtained using the sliding window (Zhou et al., 2016). The map spanned total genetic distance of 1533.72 cM with an average distance between markers of 0.33 cM (Supplementary Figure S1). The QTL locations of the *per se* performances and GCA effects for eight yield-related traits in each of four environments were determined using a composite-interval mapping (CIM) method with the *R/qtl* package (Broman et al., 2003), and a joint analysis across all environments was performed using BLUE. The threshold for identifying a significant QTL was defined by a logarithm of the odds (LOD) score of 3.0 calculated using 1000 permutations (*P* < 0.05). The QTL confidence interval was considered as genomic regions within a 1.5-LOD drop from the peak LOD scores. The proportion of phenotypic variation explained by each QTL was calculated using the *fitqtl* function in the R “*qtl*” package. QTL detected in different environments for different traits with overlapping confidence intervals or whose peaks were within 20 Mb of each other were considered as pleiotropic QTL (Frascaroli et al., 2007).

## Results

### Performance of RILs and Testcross Populations for Yield-Related Traits

**FIGURE 1:**
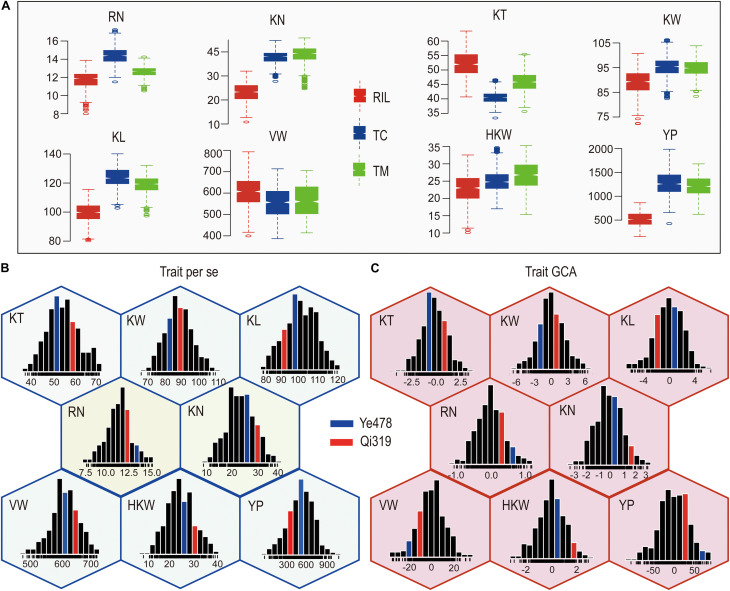
Phenotypic variation in eight traits in different populations. **(A)** The means and ranges of eight yield-related traits in the RILs and their testcross progenies. **(B,C)** Phenotypic variation of *per se* performances and their GCA effects for eight yield-related traits. The histograms inside each hexagon indicate the distribution of phenotypic values for each trait. RN, Row number; KN, Kernel number per row; KT, Kernel thickness; KW, Kernel width; KL, Kernel length; HKW, 100-kernel weight; VW, Volume weight; YP, Yield per plot.

The means and ranges of eight yield-related traits measured in the RILs and their testcross progenies were shown in Figure 1. The TC and TM values for most of the traits except for KT and VW were significantly higher than the corresponding values in the RIL population (*P* < 0.01). The percentages of testcross progenies were with higher values than RILs for these traits ranged from −22.98% (KT) to 141.46% (YP) for TC and −12.36% (KT) to 130.13% (YP) for TM. Notably, the percentages for KN and YP were 61.55% and 141.46% for TC and 66.48% and 130.13% for TM (Figure 1A), indicating that KN and YP showed apparent heterosis in these two testcross populations. In addition, percentages for KT were −22.98% for TC and −12.36% for TM, suggesting that the average performances of RILs were higher than testcross progenies for KT. The GCA effects of eight yield-related traits in RILs were normally distributed with the average around zero, and the variations for the GCA effects of YP and VW were larger than those for the GCA effects of other traits (Supplementary Table S1). The value of all the traits in the RIL population also showed a continuous and normal distribution (Figure 1B), indicating the presence of complex underlying genetic mechanisms for *per se* performances and GCA effects for yield-related traits.

### Correlation Analysis Between *per se* Performance and Corresponding GCA Effects for Yield-Related Traits and Variance Analysis of Combining Ability

The correlations between the phenotypic values of *per se* performances and the corresponding GCA effects for yield-related traits were shown in Table 1. Most of the traits in RILs were significantly correlated with each other. HKW was significantly positively correlated with KT (0.56, *P* < 0.01), KW (0.77, *P* < 0.01), and KL (0.42, *P* < 0.01), suggesting that kernel size was a major component of kernel weight. However, HKW was significantly negatively correlated with RN and KN, which indicated tradeoffs between HKW and RN or KN. YP was significantly positively correlated with all of the other traits (0.15–0.71, *P* < 0.01) except for KT (-0.37, *P* < 0.01), but especially with RN (0.41, *P* < 0.01), KN (0.71, *P* < 0.01), and KL (0.55, *P* < 0.01). The correlations between the GCA effects of YP and the GCA effects of KN, KL, and HKW were relatively strong, indicating that it was important to select inbred lines with relatively high GCA effects for these traits. In addition, significant positive correlations were observed between GCA effects and *per se* performances for all eight yield-related traits (0.17–0.64, *P* < 0.01) (Table 2), for which strong correlations were detected for RN (0.64, *P* < 0.01), KT (0.54, *P* < 0.01), KW (0.62, *P* < 0.01), and HKW (0.53, *P* < 0.01).

**TABLE 1:** Phenotypic correlation coefficients (*r*) between *per se* performances and GCA effects for eight yield-related traits.

Traits	RN	KN	KT	KW	KL	VW	HKW	YP
RN	0.64**	−0.19**	−0.19**	−0.58**	0.22**	−0.27**	−0.40**	0.06
KN	0.26**	0.34**	−0.32**	–0.09	−0.14*	0.03	−0.27**	0.38**
KT	−0.30**	−0.62**	0.54**	0.12*	−0.21**	0.14**	0.48**	0.09
KW	−0.24**	−0.15**	0.31**	0.62**	0.34**	−0.018**	0.63**	0.15**
KL	0.26**	0.20**	−0.13*	0.52**	0.44**	−0.57**	0.34**	0.44**
VW	−0.15**	0.15**	–0.10	−0.19**	−0.25**	0.38**	0.14*	–0.07
HKW	−0.25**	−0.30**	0.56**	0.77**	0.42**	0.08	0.53**	0.48**
YP	0.41**	0.71**	−0.37**	0.21**	0.55**	0.15**	0.18**	0.17**

Phenotypic correlation coefficients (r) between per se performances for eight yield-related traits are below the diagonal. Phenotypic correlation coefficients (r) between GCA for grain yield-related traits are above the diagonal. Phenotypic correlation coefficients (r) between per se performances and their GCA effects for eight yield-related traits are on the diagonal in bold font. RN, Row number; KN, Kernel number per row; KT, Kernel thickness; KW, Kernel width; KL, Kernel length; HKW, 100-kernel weight; VW, Volume weight; YP, Yield per plot. * and ** indicate significance at P < 0.05 and P < 0.01, respectively.

**TABLE 2:** Combined analyses of variance for eight yield-related traits in Ye478 × Qi319 RILs.

Source of variation	Mean square							
	RN	KN	KT	KW	KL	VW	HKW	YP
Genotype (G)	0.63**	8.10**	12.90**	15.03**	19.96**	490.44**	5.58**	6990.41**
Location (L)	0.05**	0.01**	4.04**	6.73**	15.49**	4836.74**	13.08**	9538.99**
Year (Y)	0.17**	8.43**	3.95**	0.00	7.02**	462.86**	0.30**	6703.86**
G × L	0.00	0.70**	0.00	0.16**	0.00	164.47**	0.00	840.99**
G × Y	0.02**	0.01**	0.00	0.02**	0.00	0.00	0.00	0.00
G × L × Y	0.15**	3.20**	3.29**	3.54**	10.40**	84.87**	3.89**	6226.95**
Block (B)	0.01**	0.06**	0.00**	0.30**	0.65**	1.46**	0.11**	371.81**
Error (E)	0.42**	5.58**	10.33**	13.96**	22.49**	2685.31**	7.55**	8969.73**
*H*^2^	0.86	0.81	0.86	0.85**	0.79	0.53	0.74	0.69
Coefficient of variation (CV)	0.02	0.04	0.02	0.03	0.03	0.11	0.03	0.05

* and ** indicate significance at P < 0.05 and P < 0.01, respectively. RN, Row number; KN, Kernel number per row; KT, Kernel thickness; KW, Kernel width; KL, Kernel length; HKW, 100-kernel weight; VW, Volume weight; YP, Yield per plot.

ANOVA revealed highly significant differences among genotypes, environments, and the genotype-by-environment interactions for these eight yield-related traits (Table 2). Broad-sense heritability (*H*^2^) was high for most of the yield-related traits [ranging from 0.74 (HKW) to 0.86 (RN, KT)], suggesting high heritability for these traits, except for VW and YP. The variances for GCA*_*k*_* and SCA*_*kl*_* were significant for all of the traits, suggesting that both of these genetic effects were important for controlling the inheritance of the traits (Table 3). Higher ratios of σ^2^_GCA_/σ^2^_SCA_ [1.16 (YP) to 24.56 (KT)] suggesting that the predominance of additive gene action was more important for the expression of these yield-related traits, especially for RN (17.60) and KT (24.56).

**TABLE 3:** Combined analyses of variance for eight yield-related traits in the testcross population.

Source of variation	Mean square							
	RN	KN	KT	KW	KL	VW	HKW	YP
Location (L)	0.07**	0.82**	0.16**	0.00	8.73**	6834.82**	7.70**	35837.44**
Year (Y)	0.00	4.28**	0.00	0.65**	0.00	41.48**	0.00	8264.18**
GCAk	0.21**	1.50**	2.15**	4.89**	5.91**	182.33**	1.59**	1684.67**
GCAl	1.43**	0.34**	13.77**	0.03**	8.38**	6.03**	0.00	0.04**
SCAkl	0.09**	0.83**	0.65**	0.94**	1.55**	41.24**	0.35**	1452.68**
L × GCAk	0.00	0.04**	0.13**	0.00	0.63**	13.40**	0.05**	1252.77**
L × GCAl	0.00	0.00	3.00**	0.00	0.00	25.73**	0.87**	1165.38**
L × GCAkl	0.00	0.00	0.09**	0.42**	0.23**	0.00	0.05**	0.00
Y × GCAk	0.00	0.20**	0.09**	0.00	0.21**	13.10**	0.05**	714.71**
Y × GCAl	0.00	0.00	0.00	0.00	0.00	0.00	0.87**	9873.17**
Y × GCAkl	0.01**	0.10**	0.10**	0.86**	1.42**	10.34**	0.42**	315.97**
L × Y × GCAk	0.01**	0.46**	0.03**	0.35**	0.62**	48.33**	0.46**	269.46**
L × Y × GCAl	0.04**	1.39**	0.85**	0.29**	25.08**	82.87**	2.89**	487.01**
L × Y × GCAkl	0.02**	1.02**	0.05**	0.00	0.75**	0.00	0.18**	2055.55**
Block (B)	0.00	0.09**	0.11**	0.08**	0.00	9.34**	0.01**	407.69**
Error (E)	0.38**	4.20**	4.16**	12.30**	19.98**	1333.16**	5.87**	25822.13**
GCA/SCA	17.60	2.23	24.56	5.26	9.24	4.57	4.53	1.16
*H*^2^	0.94	0.52	0.85	0.66	0.57	0.43	0.36	0.13
Coefficient of variation (CV)	0.08	0.31	0.25	0.05	0.68	0.34	0.38	0.55

* and ** indicate significance at P < 0.05 and P < 0.01, respectively. k and l indicated the kth male (RILs) and the lth female (Tester). RN, Row number; KN, Kernel number per row; KT, Kernel thickness; KW, Kernel width; KL, Kernel length; HKW, 100-kernel weight; VW, Volume weight; YP, Yield per plot.

### QTL Detection

#### QTL for *per se* Performances for Eight Grain Yield Related Traits

**FIGURE 2:**
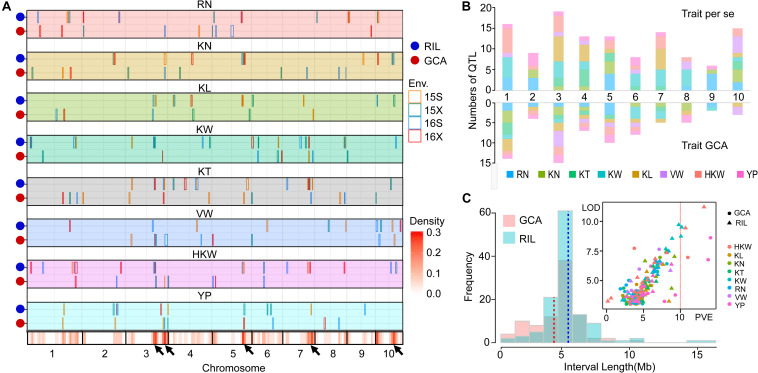
Chromosomal distributions of yield-related QTL identified in the present study. **(A)** QTL regions represented by the confidence intervals for linkage mapping across the maize genome from each dataset are shown as boxes. The width of the boxes reflects the length of the confidence interval. The different colors of the boxes indicate the four environments, respectively. *Orange*, 2015 Shijiazhuang; *green*, 2015 Xinxiang; *blue*, 2016 Shijiazhuang; and *red*, 2016 Xinxiang. The *x*-axis indicates genetic positions across the maize genome in Mb. The heatmap under the *x*-axis illustrates the density of grain yield-related related QTL across the genome. The black arrows point out the QTL hotspots. RN, Row number; KN, Kernel number per row; KT, Kernel thickness; KW, Kernel width; KL, Kernel length; HKW, 100-kernel weight; VW, Volume weight; YP, Yield per plot. **(B)** The numbers of QTL distributed on each chromosome for the eight yield-related traits identified for *per se* performances of traits (above) and their GCA effects (below). **(C)** Frequency distribution of QTL interval length and the variance explained by each QTL identified for *per se* performances of traits and their GCA effects. PVE, phenotypic variation explained.

To better understand the genetic basis of *per se* performances and GCA effects for eight yield-related traits, QTL mapping in four environments (Supplementary Tables S3, S4) and joint analyses were performed in the present study (Table 4). The results of the joint analyses showed that a total of 36 loci were identified for *per se* performances for eight yield-related traits (Table 4). A total of 121 significant QTL were associated with *per se* performances for these traits in four environments, and 26 of these QTL were detected in multiple environments (Figure 2A). The QTL were distributed over all 10 maize chromosomes, ranging from six on chromosome 9–19 on chromosome 3 (Figure 2B). Between 11 and 23 significant QTL were identified for RN, KN, KT, KW, KL, and HKW, while only 10 significant QTL were detected for VW, which might be due to the low heritability of VW. All of these QTL could individually explain between 2.46 and 12.71% of the variation in particular traits (Figure 2C). The confidence intervals for these QTL spanned physical distances ranging from 2.15 to 18.85 Mb, with an average of 5.52 Mb, with a mode for physical distance of about 5 Mb (Figure 2C). Notably, *qHKW3-2* explained the greatest proportion of phenotypic variation for HKW and co-localized with QTL for three grain morphological traits, including *qKT3-3*, *qKW3-3*, *qKL3-3*, and all of these QTL were identified in at least two environments. Qi319 alleles had positive effects on performance for KT, KL, KW, and HKW (Supplementary Table S3).

**TABLE 4:** QTL and corresponding GCA effects detected for eight yield-related traits in RILs.

Trait name^a^	Name^b^	Chr.^c^	Env.^d^	Interval (Mb)^e^	PVE^f^	ADD^g^	Env.^d^	Interval (Mb)^e^	PVE^f^	ADD^g^
RN	*qRN1-4*	1	15S/15x	221.15–237.25	5.38	0.43				
	*qRN2-1*	2	16X/C	7.70–10.85	5.52	0.41	15x/C	8.65–13.3	3.46	0.08
	*qRN2-2*	2	16S/16X/C	203.05–209.10	5.14	−0.40				
	*qRN3-1*	3					C	21.45–25.65	7.03	−0.21
	*qRN3-2*	3	C	203.65–207.5	4.23	0.29				
	*qRN4-1*	4					C	3.45–11.9	3.68	0.15
	*qRN4-3*	4					C	189.65–216.4	3.71	−0.15
	*qRN5-2*	5	15X/C	10.35–13.85	5.79	0.40	16s/C	8.75–26.2	3.46	0.06
	*qRN5-4*	5	15S/16S/16X	160.75–167.55	5.86	0.48	C	147.05–176.95	1.10	0.09
	*qRN6*	6					C	156.15–159.15	7.89	0.22
	*qRN7*	7					C	0.35–2.65	3.02	−0.14
	*qRN8-2*	8					C	166.15–169.15	2.84	−0.13
	*qRN9-1*	9	15S/15X/16S/16X/C	7.85–16.85	4.71	0.37	C	10.9–16	5.83	0.19
	*qRN10-1*	10	15S/15x	10.35–19.40	5.37	−0.43				
	*qRN10-2*	10					C	95.05–100.4	8.51	−0.23
KN	*qKN1-1*	1					C	12.55–17.55	3.81	−0.38
	*qKN1-4*	1					C	207.05–213.05	4.80	−0.43
	*qKN1-6*	1					C	270.35–275	8.43	0.57
	*qKN2*	2	15X/16X/C	171.95–183.20	4.55	−1.37				
	*qKN3-1*	3					15x/C	9.95–22.4	4.51	−0.25
	*qKN3-3*	3					C	183.35–188.5	3.48	−0.36
	*qKN5*	5	15X/16S/16X/C	165.05–183.55	4.82	1.42				
	*qKN7-1*	7					C	113.1–117.7	12.24	−0.67
	*qKN7-3*	7					C	174.45–176.65	2.73	0.32
	*qKN8-2*	8					16s/C	170.45–171.95	4.05	0.24
	*qKN10-2*	10	15X/16S/C	91.25–102.60	6.02	1.60				
KT	*qKT1-2*	1					15S/16X	203.55–208.95	5.72	0.01
	*qKT1-3*	1					C	266.35–274.4	4.12	−0.53
	*qKT2*	2					C	2.05–3.2	7.25	−0.71
	*qKT3-1*	3					C	2.45–4.8	2.00	0.39
	*qKT3-3*	3	15S/16S/C	152.70–170.25	5.43	1.74	C	154.85–160.25	2.94	0.47
	*qKT4-1*	4	15S/16S	30.00–39.90	5.93	−2.07				
	*qKT4-2*	4	15X/16X/C	128.15–134.40	6.76	−1.92	15x/C	126.5–144.75	7.63	−0.45
	*qKT5-1*	5	15S/16X	166.15–173.10	5.28	−1.89				
	*qKT5-2*	5					15x/C	203.7–210.8	2.47	0.21
	*qKT6*	6	15X/C	1.30–10.40	4.30	−1.53				
	*qKT7-1*	7	15S/C	115.35–134.05	4.25	1.42	C	113.1–118.9	5.50	0.63
	*qKT8*	8					C	147.05–159.75	2.17	0.39
	*qKT9*	9					C	16.2–18.45	2.76	−0.43
	*qKT10-1*	10					C	18.1–23.6	4.56	−0.56
KW	*qKW1-1*	1	15X/16S/16X/C	19.25–35.35	6.89	−2.28				
	*qKW1-3*	1	15S/16S	246.90–274.65	3.27	−1.75	C	262.9–268.25	5.56	−0.92
	*qKW3-1*	3					C	18.85–27.8	0.28	0.29
	*qKW3-2*	3					C	36.35–49.85	0.01	0.07
	*qKW3-3*	3	15X/16S/16X/C	155.05–165.40	8.75	2.64	C	149.65–157.55	1.37	0.59
	*qKW4-1*	4					C	26.3–43.9	0.35	0.41
	*qKW4-2*	4					C	58.15–130.7	0.13	0.25
	*qKW4-3*	4	16S/C	173.50–210.70	2.19	1.24	C	195.2–201.1	4.78	0.86
	*qKW5-1*	5					C	63.8–90.65	0.06	−0.16
	*qKW5-2*	5					C	119.7–154.8	0.64	−0.54
	*qKW6-4*	6					15x/C	140.7–149.65	8.69	−0.65
	*qKW6-5*	6					16x/C	164.75–168.75	4.31	−0.46
	*qKW7-2*	7	15S/15X/16X/C	126.10–148.90	6.97	2.20	C	127.05–131.1	6.32	0.98
	*qKW8*	8	15s/15 × 16S/16X/C	161.90–171.35	5.48	−1.99				
	*qKW9*	9					15x/C	2.7–16.2	3.24	−0.31
	*qKW10*	10	16S/C	100.40–116.80	5.66	−2.02				
KL	*qKL1-1*	1					C	88.2–148.65	2.42	−0.67
	*qKL1-2*	1					16x/C	191.75–202.6	4.44	−0.53
	*qKL2*	2	C	1.50–3.65	3.20	1.48	15s/C	2.05–10.05	7.01	0.74
	*qKL3-2*	3	15S/C	34.80–51.95	6.05	2.63				
	*qKL3-3*	3	15X/16S/16X	159.05–165.8	5.94	3.14				
	*qKL3-4*	3	15S/16S/C	209.10–217.25	7.84	2.72	16x/C	204.25–230.45	4.07	0.47
	*qKL4-1*	4					C	30.25–36.2	5.91	1.00
	*qKL4-3*	4	15S/C	152.25–159.20	5.19	2.25				
	*qKL5-2*	5					C	204.35–206.65	3.59	−0.77
	*qKL7-2*	7	15S/15X/16X/C	137.05–151.50	6.19	2.53				
	*qKL9*	9					C	106.1–134.25	2.60	0.66
	*qKL10-2*	10	15X/C	82.55–118.65	3.59	−2.01	C	91.25–97.7	9.50	−1.27
VW	*qVW3-3*	3					15x/16x	161–171.25	1.76	0.75
	*qVW4-1*	4					C	31–36.7	6.73	−5.51
	*qVW5-2*	5					C	187.2–217.35	2.62	−3.42
	*qVW6-1*	6					15x/C	0.35–4.1	5.58	3.38
	*qVW10-1*	10	15X/16S/C	4.05–34.60	4.26	13.77				
	*qVW10-2*	10					15S/C	100.4–108.35	9.31	4.44
HKW	*qHKW1-1*	1	16X/C	19.85–26.85	5.84	−1.43				
	*qHKW1-2*	1	15X/16S	86.30–93.15	4.04	−1.45				
	*qHKW1-3*	1	15S/16S/16X/C	249.15–277.15	4.51	−1.14	16x/C	262.9–271.2	10.78	−0.46
	*qHKW2-1*	2	C	6.05–9.65	4.68	−0.94				
	*qHKW3-1*	3					C	16.9–22.4	2.74	0.37
	*qHKW3-2*	3	15S/15X/16S/16X/C	156.45–167.6	8.89	1.64	15x/C	142.55–171.25	5.57	0.26
	*qHKW4*	4					15x/C	1.55–6.1	4.34	−0.26
	*qHKW5-1*	5					16s/16x	4.95–21.6	5.12	0.12
	*qHKW5-2*	5	15X/15S/C	164.95–184.45	4.36	−1.21				
	*qHKW6-2*	6					C	155.15–168.75	3.27	−0.35
	*qHKW7-2*	7					C	126.1–131.55	7.49	0.53
	*qHKW7-3*	7	15S/16X/C	138.55–153.95	8.01	1.56				
	*qHKW8*	8	16X/C	161.35–166.15	4.14	−1.12				
	*qHKW9*	9					C	9.55–17.25	2.51	−0.31
	*qHKW10*	10	15X/16S/C	82.00–118.90	4.76	−1.44				
YP	*qYP1*	1	15S/C	201.95–207.95	4.04	−37.90	15S/C	196.5–201.1	7.38	−12.41
	*qYP2-3*	2	16S/16X/C	190.40–199.05	5.73	−50.69				
	*qYP5-2*	5					15S/16x	174.45–214.45	3.54	−4.96
	*qYP9-1*	9	16S/C	98.10–105.55	5.13	43.46				
	*qYP9-2*	9					C	149.15–153.55	5.23	13.86
	*qYP10*	10					C	80.35–85.7	9.18	−18.62

**^*a*^Trait refers to the name of each component of yield-related traits: RN, Row number; KN, Kernel number per row; KT, Kernel thickness; KW, Kernel width; KL, Kernel length; HKW, 100-kernel weight; VW, Volume weight; YP, Yield per plot. ^*b*^The name of each QTL is a composite of the influenced trait: RN, KN, KT, KW, KL, HKW, VW, or YP. ^*c*^Chr, chromosome. ^*d*^QTL in a specific environment: 15S is 2015 Shijiazhuang; 15X is 2015 Xinxiang; 16S is 2016 Shijiazhuang; 16X is 2016 Xinxiang; C represents joint analyses. ^*e*^Interval, confidence interval between two bin markers. ^*f*^PVE, the phenotypic variance explained by an individual QTL. ^*g*^ADD, the value of additive genetic effect. LOD scores, PVE values, and ADD values are shown as mean values for QTL with multiple effects.**

#### QTL for GCA Effects of Eight Grain Yield-Related Traits

Joint analyses identified a total of 64 loci associated with GCA effects of eight grain yield-related traits. A total of 74 significant QTL for eight yield-related traits were associated with GCA effects in four environments, but only four of them were detected in multiple environments (Figure 2A). These QTL were distributed over all 10 maize chromosomes, and two (chromosome 9) to 15 (chromosome 3) QTL were detected on each chromosome. Seven to 11 significant QTL for GCA effects were identified for each trait (Figure 2B). All of these QTL could individually explain from 0.04 to 9.10% of the observed variation in a single environment (Figure 2C). The confidence intervals for these QTL spanned physical distances from 0.70 to 14.55 Mb, with an average of 4.72 Mb, a mode near 5 Mb (Figure 2C). The two QTL *qKN7-1* and *qHKW1-3* could explain more than 10% of the variation in GCA effects for KN and HKW, respectively (Table 4). Notably, the stable QTL *qHKW1-3* explained 10.78% of the phenotypic variation in the GCA effects of HKW and co-localized with the QTL for the GCA effects of four grain morphological traits: *qKT1-3*, *qKW1-3*, *qKL1-4*, and *qKN1-6*. Qi319 alleles had a negative effect on KT, KW, KL, and HKW GCA effects, but had a positive effect on GCA effects for KN. These results also confirmed the close genetic correlations observed between the GCA effects of grain morphological traits, which might result from pleiotropy.

#### Comparison of the Genetic Basis Between *per se* Performances and GCA of Traits

**FIGURE 3:**
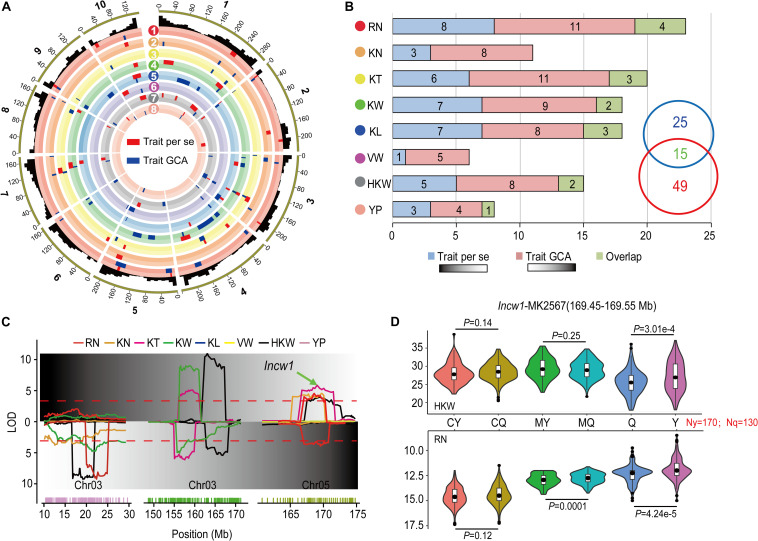
Comparison of the QTL distributions associated with *per se* performances and their GCA effects for eight yield-related traits. **(A)** Integrated QTL on the 10 maize chromosomes for eight yield-related traits across multiple environments and the joint analyses. RN (*red*), Row number; KN (*orange*), Kernel number per row; KT (*yellow*), Kernel thickness; KW (*green*), Kernel width; KL (*blue*), Kernel length; VW (*purple*), Volume weight; HKW (*gray*), 100-kernel weight; YP (*pink*), Yield per plot. The red and blue boxes indicate the QTL results in *per se* performances and their GCA effects for the traits, respectively. The width of the boxes reflects the length of the confidence intervals for each QTL. **(B)** QTL numbers distributed on each dataset for eight yield-related traits across multiple environments and joint analyses. Green boxes show the number of QTL overlapping between *per se* performances and their GCA effects for the traits. **(C)** A typical pleiotropic locus on chromosomes 3 and 5. The *x*-axis indicates the genetic positions across the maize genome in Mb. The *y*-axis indicates the LOD score of the detected QTL. The green arrow shows the position of *Incw1*, which influences HKW. **(D)** Tests for differences among phenotypic values for HKW and RN associated with MK2567 in the parental inbred lines, Ye478 and Qi319, of RILs and the corresponding hybrids obtained by crossing with testers. CY and CQ represent the phenotypic values of the hybrids of Chang7-2 × Ye478 and Chang7-2 × Qi319. MY and MQ represent phenotypic values of the hybrids of Mo17 × Ye478 and Mo17 × Qi319. Y and Q represent phenotypic values of Ye478 and Qi319. *P* < 0.05 represents the level of significance for differences in the performance HKW (above) and RN (below) in CY and CQ, MY and MQ, and Y and Q, respectively.

In order to improve the reliability of our results, only the loci identified in multiple environments and the results of the joint analyses were used for subsequent analysis. A total of 95 QTL were identified that affected the *per se* performances and corresponding GCA effects for eight yield-related traits in Figure 3A and Table 4. A total of 17 of these QTL were detected for both *per se* performances and corresponding GCA effects for these traits. Zero (KN, VW) to four (RN, KW) QTL were detected for both *per se* performances and corresponding GCA effects of the traits. The numbers of QTL that co-localized for *per se* performances and corresponding GCA effects of traits corresponded to the results of correlation analysis (Tables 1, 4). For example, *qHKW3-2*, which associated with KT, KW, and HKW, was identified for both *per se* performances and corresponding GCA effects for the traits. In addition, the direction of the parental contribution was identical for the 17 co-localized QTL for the *per se* performances and corresponding GCA effects for these traits (Figure 3B). This result validated the positive correlation between the genetic basis of *per se* performances and corresponding GCA effects for these traits.

Of the 27 and 51 QTL were characteristically detected in the *per se* performances and corresponding GCA effects of traits, respectively. Two QTL, *qRN3-1* and *qRN6*, together explained 14.92% of the observed variation for the GCA effect of RN (Table 4). *qHKW3-1* was associated with GCA effects of HKW, KW, and KN. However, no significant loci were detected for the respective *per se* performances of the traits (Figure 3C). In addition, *qKT1-2* and *qHKW5-1* explained 5.72 and 5.12% of the observed variation for GCA effects of KT and HKW individually rather than *per se* performances for the traits in multiple environments. In contrast, the stable *qHKW5-2* locus that was associated with RN, KN, KT, and HKW *per se*, was only associated with the GCA effects of RN and explained only 1.10% of the observed variation in the GCA effects of RN. The results reflected the distinct genetic bases of *per se* performances and corresponding GCA effects for these traits.

The maize gene annotation database at MaizeGDB^2^ was used to predict candidate genes in the *qHKW5-2* region. *Incw1*, which encodes cell-wall invertase in the developing endosperm of maize, was located in the region surrounding MK2567 (169.45–169.55) at the *qHKW5-2* locus (Figure 3D). SNPs between Ye478 and Qi319 located around MK2567 were calculated and categorized according to parental allele. A total of 170 SNPs match the Ye478 parental genotype and 130 match the Qi319 genotype. The phenotypic values for HKW in the Ye478 × Qi319 RIL population differed significantly (*P* = 3.01e-4). Nevertheless, there was no significant difference in HKW between either the Chang7-2 × Ye478 and Chang7-2 × Qi319 F_1_ hybrid groups (*P* = 0.14) or the Mo17 × Ye478 and Mo17 × Qi319 F_1_ hybrid groups (*P* = 0.25). There were significant phenotypic differences in RN in the RIL population (*P* = 4.24e-5). However, there was no significant difference in phenotypic values for RN between the Chang7-2 × Ye478 and Chang7-2 × Qi319 F_1_ hybrid groups (*P* = 0.12), but there was a significant difference in phenotypic values of RN between Mo17 × Ye478 and Mo17 × Qi319 F_1_ hybrid groups (*P* = 0.0001).

#### Pleiotropy of Quantitative Trait Loci for Eight Grain Yield-Associated Traits

The results of single environment analysis in the present study showed that five QTL hotspots were located on chromosomes 3, 5, 7, and 10 (Figure 2A). Integrating the results of QTL detected in multiple environments and during the joint analyses indicated that the most densely distributed QTL were highly concentrated in several chromosomal regions on chromosomes 1, 2, 3, 7, and 10 (Figure 3A). These QTL hotspots were likely responsible for pleiotropy. The non-random distribution of these pleiotropic loci might explain the correlation between some of these traits.

A total of 11 pleiotropic loci were detected for *per se* performances of the traits, and five, two, and four loci were simultaneously associated with two, three, and four traits, respectively, in the RILs. Loci affecting four traits each were located on chromosome 3, 5, 7, and 10, respectively. For instance, *qHKW3-2* and *qHKW7-3* were simultaneously associated with KW, KT, KL, and HKW. These results suggested that the *qHKW3-2* and *qHKW7-3* loci might affect grain yield via the development of grain morphology. Further, the *qHKW5-2* locus was simultaneously associated with RN, KN, KT, and HKW. These results suggested that *qHKW5-2* might affect grain yield by exerting effects on the differentiation and development of maize ears (Table 4). A total of 16 pleiotropic loci were identified that were associated with GCA effects, with four, five, and seven loci were simultaneously associated with the GCA effects of two, three, and four traits. These loci that were simultaneously detected for four traits were located on chromosomes 1, 3, 5, 7, 9, and 10, respectively. In general, these pleiotropic loci were associated with the GCA effects of YP or HKW. Most of the pleiotropic loci were detected for both *per se* performances and corresponding GCA effects of traits. Notably, *qHKW10*, which was associated with eight traits, was detected for the *per se* performances for the traits KN, KL, KW, and HKW *per se*, but was associated with the GCA effects of KL, RN, VW, and YP. This suggested that the genetic bases of these traits in RILs and their corresponding GCA effects could either be similar or different. Thus, these pleiotropic loci could be used to improve the GCA of traits while also simultaneously improving per se performances for yield-related traits.

## Discussion

### Comparative Mapping of QTL for Yield-Related Traits in the Spring and Summer Maize-Growing Region of China

The present QTL analysis of yield-related traits in the same population in different areas of the maize-growing region in China was helpful for adequately exploring the genetic basis of yield-related traits in maize. Earlier, the identical RIL population was planted in 2013 and 2014 in the spring maize-growing regions of Beijing and Gongzhuling, China (Zhang et al., 2017). Those results revealed three stable QTL associated with HKW located on chromosomes 1, 7, and 9, respectively. Among those loci, the *qHKW7* locus located on chromosome 7 from position 133.60 to 139.25 Mb, explained the greatest proportion of phenotypic variation (7.13%) for HKW in this population and environment. We also found that *qHKW7* was a polymorphic QTL that also influences kernel size. In the present study, the same RILs were planted in the summer maize-growing region of Shijiazhuang and Xinxiang, China, in 2015 and 2016. About 41.27% of the QTL detected in the present study were consistent with those identified in the previous study for six yield-related traits (Supplementary Table S3). Here, the major QTL *qHKW3-2* with the major effects on HKW was located on chromosome 3 from position 156.45–167.6 Mb. Other significant QTL related to HKW were also detected on chromosomes 1 and 7, in the same intervals as in our previous studies, except for the *qHKW9* locus. The current results showed that the loci on chromosomes 1 and 7 were the main QTL controlling HKW in both spring and summer maize-growing regions. However, the QTL on chromosome 3 only affected HKW in the summer maize-growing region, and that on chromosome 9 only affected HKW in the spring maize-growing region. The spring maize-growing region has a shorter maize growing season than the summer maize-growing region does, and growing season length was significantly correlated with HKW. This difference might explain the distinct genetic effects on yield-related traits in the spring and the summer maize-growing regions in the present study. *qRN3-1*, a major QTL that explained 7.03% of the observed variation in the GCA effects of RN, could not be detected for *per se* performances of the traits in both of these maize-growing regions. Thus, MAS for *qRN3-1* will also be promoted for breeding hybrid combinations with optimal row number.

### Correlation Between the Basis of General Combining Ability and *per se* Performances and GCA for Yield-Related Traits

Parental inbred lines with high combining ability were considered essential for the superior performance of hybrids (Duvick et al., 2004). Although GCA effects might be predicted based on the yield performance of inbred lines, the correlation between the yield performance of inbreds and that of their hybrid progeny was still not adequate for direct selection of inbred lines with high combining ability based on the *per se* yield of RILs (Lv et al., 2012). In general, although the correlations between the yield performance of inbred lines and their corresponding GCA effects in maize are positive, they are generally not strong. Previous studies have suggested that GCA effects were not significantly (*r* ≤ 0.44) correlated to their corresponding GCA effects for the yield-related traits YP, RN, KN, HKW, and PH (Huang et al., 2013). Similar results were found with another set of ILs whose performance for GCA effects were weakly (-0.01 ≤ *r* ≤ 0.49) correlated to the *per se* performances for the traits YP, KN, HKW, EL, PH, and EH. However, strong correlations have been detected between RN and its corresponding GCA effects (Qi et al., 2013). Positive and strong (0.55 ≤ *r* ≤ 0.77) correlations were identified between the three plant height related traits PH, EH, and IN in RILs (Zhou et al., 2018). These results suggest that correlations between traits have been influenced by genetic selection during the gathering of germplasm genetic resources and by environmental selection in planting locations.

In the present study, performances of GCA effects were significantly correlated to the *per se* performances of RILs for all yield-related traits (0.17 ≤ *r* ≤ 0.64, *P* > 0.01). The correlation between RN and its GCA effects was the strongest (*r* = 0.64), and was consistent with the results of previous studies (Qi et al., 2013). However, the performances of GCA effects were also strongly correlated to the *per se* performances for the traits KT, KW, and HKW. This agreed well with the results of QTL co-localization. The consistent genetic basis of the traits could be used to improve the GCA while improving the *per se* performances for these traits. However, the correlations between GCA effects and their corresponding *per se* performances for the traits KN, KL, VW, and YP were weak and indicated different genetic bases of the *per se* performances and their corresponding GCA effects for these traits. Due to diverse genetic backgrounds, various QTL populations can perform differently for specific traits. The selection of testers affected the performance of testcrosses directly, and was essential for the evaluation of GCA effects and the selection of elite inbred lines (Zhou H. et al., 2017). Non-genetic components, such as the environmental sensitivities of quantitative traits, can also affect phenotypes. Thus, it is necessary to study the genetic basis of quantitative traits and combining ability in multiple environments for many years with multiple populations.

### The Reliability and Validity of QTL for GCA Effects

GCA was generally estimated by the method of variance analysis based on diallel crossing design (Griffing, 1956). With a large number of inbred lines, the huge workload was generated by the design of diallel cross design. In contrast, combinations with high GCA and parental lines with high SCA could be identified with ease by utilizing mating designs like incomplete diallel crossing or NCII (Comstock and Robinson, 1948), and the efficiency was similar to that of diallel crossing design (Dhillon and Singh, 1978). In rice, QTL analyses for 10 agronomic traits were conducted in a backcross recombinant inbred lines population and three testcross lines, and the results indicated similarities between the genetic characteristics of *per se* performances and corresponding GCA effects for these traits (Qu et al., 2012). Combining abilities for seven bioenergy and biomass related traits were predicated by 285 diverse dent inbred lines crossing with two flint testers. The prediction accuracies ranged from 0.60 to 0.80 for metabolites and 0.72–0.81 for SNPs with metabolic and whole-genome prediction models (Riedelsheimer et al., 2012). 365 F_11_ RILs using genotyping by a high-density linkage map with 4602 high-quality bin markers in the present study had provided the precision for QTL (Zhou et al., 2016). In the previous study, the power of QTL detection for grain yield and other agronomical important traits were estimated in maize using two independent samples (*N* = 107 and 344) of F_2_ plants with two testers in four environments. A total of 39 QTL with *N* = 107 and 107 QTL with *N* = 344 were detected for all traits and both testers. It also showed that QTL accounting for at least 10.2% of phenotypic variation in Experiment 2 (*N* = 107) could be detected, but as little as 3.3% of phenotypic variation in Experiment 1 (*N* = 344) with a LOD threshold of 2.5 (Melchinger et al., 1998). A set of 365 RILs and two testers were analyzed in the present study. The genetic bases of GCA for eight yield-related traits were dissected using a hybrid panel composed of 656 hybrids obtained by 328 RILs, which were successfully crossed both with Chang7-2 and Mo17. The amount of progeny was sufficient to identify more minor QTL for *per se* performances and corresponding GCA effects for the traits. Identification of marker-QTL associations also depended upon the magnitude of allele contrasts (Kerns et al., 1999). Consistency of QTL effects across testers was in agreement with corresponding genotypic correlations between the two testcross series (Melchinger et al., 1998). The power of combining ability detection can be improved by using the elite inbred lines in the opposite heterosis group as the tester (Lu et al., 2009). The influence on GCA effect estimation caused by testers can be eliminated by using standard testers. The two testers were Chang7-2 and Mo17, which belong to the SPT and Lancaster heterotic groups, respectively. Both of the testers were elite inbred lines and used widely in commercial maize breeding. Thus, the two testers in present study could provide abundant favorable alleles for identified variety QTL for traits GCA effects. In addition, the QTL × Environment interactions contributed to the lack of congruency of QTL found for multiple experiments (Melchinger et al., 1998; Kramer et al., 2009). In present study, the specific QTL locations of the *per se* performances and corresponding GCA effects for yield-related traits in each of four environments were determined (Supplementary Tables S3, S4), and a joint analysis across all environments was performed using BLUE. In order to improve the reliability of our results, only the loci identified in multiple environments and the results of the joint analyses were used for comparing the genetic bases of *per se* performances and corresponding GCA effects for the traits (Table 4). Therefore, the QTL identified in the present study for *per se* performances and corresponding GCA effects for eight yield-related traits could be reliable and efficient for marker-assisted selection (MAS) in breeding.

### Potential Utilization of Main QTL for GCA Effects in Maize Hybrid Breeding

The genetics of combining ability were complex and greatly influenced by environments (Rojas and Sprague, 1952; Walejko and Russell, 1977). Marker-assisted selection offers an efficient way to dissect the genetic basis of combining ability (Yousef and Juvik, 2001; Eathington et al., 2007). Heterosis in F_1_ was caused by combination of different alleles at a specific locus of crossing parents (Liu et al., 2012; Zaid et al., 2017; Chen et al., 2019). Although some studies on the application of heterosis and combining ability have been published in several crops such as tomatoes (Kalloo et al., 1974), soybeans (Cerna et al., 1997), wheat (Singh et al., 2004), rice (Joshi et al., 2001), and maize (Ertiro et al., 2017), only few such studies related to the genetic dissections of combining abilities have been reported in maize (Gu, 2007; Lv et al., 2012; Qi et al., 2013; Huang et al., 2013; Giraud et al., 2017; Zhou et al., 2018). Comparing the genetic basis of *per se* performances and corresponding GCA effects for yield-related traits could improve the yields of maize inbred lines without affecting their corresponding combining ability.

In the present study, we detected some loci that only influence *per se* performances or their corresponding GCA effects for the traits. For instance, *Incw1*, which encodes a cell wall invertase in the developing endosperm of maize, was confirmed to have conserved influence on seed weight in *Arabidopsis* and maize (Liu et al., 2017). *AtcwINV1*, *OsGIF1*, and *Mn1*, which have similar functions as *Incw1*, were also associated with grain yield improvement (Li B. et al., 2013). *Incw1* was identified at the stable *qHKW5-2* locus in the present study. The *qHKW5-2* locus, which was associated with *per se* performances for traits RN, KN, KT, and HKW traits *per se*, was only associated with the GCA effects of RN and was considered a minor QTL for that trait. Our results indicated that the *Incw1* gene might affect the 100-grain weight through its effects on differentiation and development of maize ears.

*qRN3-1* and *qRN6*, two pleiotropic loci, were associated with the GCA effects of RN, KW, and HKW rather than these *per se* performances for the traits. The two loci were not been identified in previous study (Qi et al., 2013), the specificities in additive effects might be caused by diverse testers (Qu et al., 2012), the identified loci occurred due to the presence of potential GCA coverage in the potential genomic regions (Zaid et al., 2019). Furthermore, *qKT1-2* and *qHKW5-1* were two stable loci for GCA effects of KT and HKW respectively, but no significant loci were detected for corresponding *per se* performances for the traits in multiple environments. This suggested that some genes or loci differ as to *per se* performances and/or corresponding GCA effects for these traits. Previous study also revealed the different genetic basis between *per se* performances and corresponding GCA effects for the traits (Eltahawy et al., 2020). Due to heterotic effects, differences between individuals in the testcross population were significantly reduced. In addition, the maternal genetic effects due to the selection of testers strongly influenced the variation in F_1_ performance and population structure (Chen et al., 2019). These factors might explain why some loci could only be detected in a single dataset. *qKT1-2* and *qHKW5-1* explained only 5.72 and 5.12% of the observed variation for GCA effects of KT and HKW individually. Previous studies suggested that traits GCA effects were mainly related with poly-genes rather than with major gene controlling the *per se* performances for the traits (Basava et al., 2019; Eltahawy et al., 2020), further loci associated with GCA effects should be explored in maize.

Some previous studies have shown that *per se* performances and corresponding GCA effects for the traits shared the same set of genetic loci. Nevertheless, the location and number of dominant QTL for GCA effects were affected by the allele frequency of testers (Austin et al., 2000; Frascaroli et al., 2009). The performances of GCA effects were also strongly correlated to the *per se* performances for the traits KT, KW, and HKW. This agreed well with the results of QTL co-localization. In addition, the QTL *qKN7-1* and *qHKW1-3*, which could explain more than 10% of the variation in the GCA effects of KN and HKW, were also detected for *per se* performances for these traits. The direction of the parental contribution was identical. In a previous study, four QTL (*qEH1-2*, *qEH5*, *qEH6-2*, and *qEH9-2*) were simultaneously detected in *per se* performances and corresponding GCA effect, and together explained 18.16 and 19.70% of the observed variation for EH and EH GCA effects (Zhou et al., 2018). Therefore, the application of phenotypic or maker-assisted selection for improving inbred lines and GCA effects simultaneously could be evaluated economically and effectively (Mihaljevic et al., 2005; Qu et al., 2012).

## Conclusion

In conclusion, we have compared the genetic basis of *per se* performances and corresponding GCA effects for eight grain yield-related traits. A total of 95 QTL were identified that affected *per se* performances and corresponding GCA effects for eight yield-related traits, and 17 of these QTL were detected for both *per se* performances and corresponding GCA effects of traits in multiple environments and the results of the joint analyses datasets. The genetic characteristics of the traits GCA effects were consistent or inconsistent with *per se* performances for the traits. Therefore, the congruous and diverse QTL identified in the present study for *per se* performances and corresponding GCA effects for yield-related traits should be effective for maize hybrid breeding.

## Data Availability Statement

The datasets generated for this study can be found in the http://www.ncbi.nlm.nih.gov/bioproject/627044, Accession: PRJNA627044.

## Ethics Statement

We claim that the experiments described herein comply with the ethical standards in China. Informed consent was obtained from all individual participants included in the study.

## Author Contributions

XLu and ZZ performed the experiments and wrote the manuscript. ZY, CZ, ZH, ZW, ML, DZ, HY, and JH performed the experiments and revised the manuscript. XLi and JW designed the experiments. All authors contributed to the article and approved the submitted version.

## Conflict of Interest

The authors declare that the research was conducted in the absence of any commercial or financial relationships that could be construed as a potential conflict of interest.

## Footnotes

1 http://www.ncbi.nlm.nih.gov/bioproject/627044

2 http://www.maizegdb.org

## Abbreviations

GCA: general combining ability
HKW: 100-kernel weight
KL: Kernel length
KN: Kernel number per row
KT: Kernel thickness
KW: Kernel width
QTL: quantitative trait loci
RILs: recombinant inbred lines
RN: row number
SCA: specific combining ability
VW: volume weight
YP: yield per plot.
