# Supplementary material for: Genetic Dissection of the General Combining Ability of Yield-Related Traits in Maize
Source: Front Plant Sci. 2020 Jul 22;11:788. doi: 10.3389/fpls.2020.00788 (PMC7387702; doi:10.3389/fpls.2020.00788)
Supplement: Supplementary file 2 [file Data_Sheet_1.docx]

**Supplementary Table S1** Phenotypic values for traits and their GCA effects in the RILs across four environments and in joint analyses

| Trait^a^ | Env^b^ | RILs |  |  |  | GCA |  |  |
| --- | --- | --- | --- | --- | --- | --- | --- | --- |
|  |  | Range | Skew | Kurt |  | Range | Skew | Kurt |
| RN | 15S | 9.25 to 14.63 | -0.3 | 1.41 |  | -0.04 to 0.05 | 0.68 | 0.64 |
|  | 15X | 8.50 to 15.00 | -0.27 | 1.25 |  | -0.04 to 0.05 | 0.18 | 0.67 |
|  | 16S | 8.00 to 13.63 | -0.43 | 0.27 |  | -0.04 to 0.05 | 0.10 | 1.33 |
|  | 16X | 8.13 to 14.38 | -0.3 | 0.27 |  | -0.03 to 0.04 | 0.22 | -0.32 |
|  | C | 7.79 to 15.96 | 0.02 | 0.29 |  | -1.02 to 1.15 | 0.2 | 0.08 |
| KN | 15S | 14.38 to 35.13 | -0.15 | 0.81 |  | -1.13 to 1.15 | 1.65 | 1.45 |
|  | 15X | 10.57 to 35.63 | -0.25 | 0.09 |  | -1.03 to 0.83 | -0.41 | 0.43 |
|  | 16S | 10.82 to 32.13 | 0.21 | -0.08 |  | -1.14 to 1.56 | -0.13 | 0.25 |
|  | 16X | 14.00 to 33.44 | 0.2 | -0.28 |  | -0.91 to 0.84 | -0.11 | 0.09 |
|  | C | 10.93 to 38.25 | 0.09 | -0.31 |  | -2.74 to 2.72 | 0.11 | -0.04 |
| KT | 15S | 38.24 to 67.21 | 0.67 | 1.62 |  | -0.39 to 0.29 | 0.73 | 0.43 |
|  | 15X | 41.24 to 72.07 | 0.6 | 1.02 |  | -0.40 to 0.54 | 0.23 | 0.71 |
|  | 16S | 41.11 to 69.78 | 0.33 | 0.12 |  | -0.39 to 0.39 | 0.05 | 0.64 |
|  | 16X | 43.14 to 74.22 | 0.51 | 0.61 |  | -0.50 to 0.45 | 0.11 | 0.98 |
|  | C | 36.36 to 68.09 | 0.16 | -0.28 |  | -3.99 to 3.64 | 0.05 | 0.12 |
| KW | 15S | 72.38 to 101.33 | -0.06 | -0.1 |  | -0.34 to 0.32 | 0.07 | 0.01 |
|  | 15X | 76.23 to 107.45 | 0.09 | 0.31 |  | -0.35 to 0.30 | 0.00 | 0.37 |
|  | 16S | 72.27 to 104.60 | 0.27 | 0.59 |  | -0.31 to 0.45 | 0.18 | 0.63 |
|  | 16X | 75.84 to 107.43 | 0.18 | 0.04 |  | -0.41 to 0.63 | 0.55 | 0.63 |
|  | C | 68.28 to 110.58 | 0.11 | -0.13 |  | -5.46 to 5.29 | 0.03 | 0.21 |
| KL | 15S | 80.23 to 116.33 | -0.04 | 0.42 |  | -1.24 to 1.03 | 0.29 | 0.19 |
|  | 15X | 90.64 to 123.01 | -0.06 | -0.14 |  | -1.08 to 0.85 | -0.35 | 0.82 |
|  | 16S | 81.79 to 115.46 | -0.1 | 0 |  | -1.33 to 1.06 | -0.22 | 0.98 |
|  | 16X | 81.05 to 122.51 | 0.24 | 0.02 |  | -1.65 to 1.19 | -0.28 | 1.05 |
|  | C | 78.26 to 122.13 | -0.07 | -0.4 |  | -5.52 to 6.12 | -0.18 | 0.12 |
| VW | 15S | 353.83 to 669.88 | -0.3 | 0.55 |  | -7.00 to 7.77 | 0.53 | 0.45 |
|  | 15X | 492.27 to 824.91 | 0.59 | 0.79 |  | -9.89 to 10.65 | 0.38 | 0.62 |
|  | 16S | 404.62 to 705.43 | -0.18 | 0.56 |  | -9.85 to 11.43 | 0.28 | 0.75 |
|  | 16X | 538.85 to 771.53 | -0.11 | 0.36 |  | -7.08 to 8.20 | 0.22 | 0.44 |
|  | C | 502.15 to 713.78 | -0.01 | -0.2 |  | -29.97 to 30.01 | -0.15 | 0.35 |
| HKW | 15S | 10.13 to 30.09 | 0.31 | 0.7 |  | -0.88 to 0.73 | 0.33 | 0.22 |
|  | 15X | 17.90 to 41.81 | 0.48 | 0.33 |  | -0.89 to 1.03 | 0.13 | 0.12 |
|  | 16S | 12.66 to 31.17 | 0.05 | -0.06 |  | -0.68 to 0.86 | 0.08 | 0.51 |
|  | 16X | 15.92 to 36.57 | 0.2 | -0.15 |  | -1.23 to 0.95 | -0.23 | 1.36 |
|  | C | 10 to 36.53 | 0.11 | -0.2 |  | -2.93 to 2.6 | -0.04 | 0.08 |
| YP | 15S | 158.24 to 774.33 | -0.01 | -0.28 |  | -45.47 to 38.25 | 0.72 | 0.61 |
|  | 15X | 227.30 to 1126.48 | -0.26 | 1.18 |  | -44.84 to 49.17 | 0.23 | 0.78 |
|  | 16S | 154.46 to 843.60 | 0.13 | -0.17 |  | -46.41 to 43.03 | 0.01 | 0.25 |
|  | 16X | 166.25 to 978.21 | 0.03 | -0.35 |  | -56.53 to 42.84 | -0.28 | 1.25 |
|  | C | 73.64 to 948.39 | -0.25 | 0 |  | -89.5 to 77.16 | -0.15 | 0.01 |

^a^Trait refers to the name of each component of yield-related traits: RN, Row number; KN, Kernel number per row; KT, Kernel thickness; KW, Kernel width; KL, Kernel length; HKW, 100-kernel weight; VW, Volume weight; YP, Yield per plot

^b^Specific growing environments: 15S is 2015 Shijiazhuang; 15X is 2015 Xinxiang; 16S is 2016 Shijiazhuang; 16X is 2016 Xinxiang; C represents joint analyses

**Supplementary Table S2** Performance of testcross populations in different environments

| Trait^a^ | Env^b^ | TC | | |  | TM | | |
| --- | --- | --- | --- | --- | --- | --- | --- | --- |
|  |  | Range | Skew | Kurt |  | Range | Skew | Kurt |
| RN | 15S | 12.13 to 16.88 | 0.11 | 0.05 |  | 11.50 to 14.42 | 0.46 | -0.21 |
|  | 15X | 11.50 to 17.38 | -0.02 | 0.35 |  | 11.63 to 14.38 | 0.34 | -0.47 |
|  | 16S | 12.00 to 15.88 | -0.13 | -0.54 |  | 10.50 to 14.25 | 0.01 | 0.91 |
|  | 16X | 12.00 to 17.31 | 0.13 | 0.24 |  | 11.50 to 14.25 | 0.38 | 1.42 |
| KN | 15S | 29.31 to 44.81 | -0.24 | 1.5 |  | 34.94 to 46.63 | -0.23 | -0.33 |
|  | 15X | 29.38 to 44.81 | -0.58 | 1.4 |  | 31.25 to 46.94 | -0.48 | 0.34 |
|  | 16S | 27.75 to 41.00 | -0.12 | 0.27 |  | 24.63 to 45.69 | -0.35 | 0.35 |
|  | 16X | 31.56 to 43.25 | -0.1 | 0.27 |  | 25.00 to 45.31 | -0.39 | 1.42 |
| KT | 15S | 35.65 to 46.76 | 0.14 | 0.27 |  | 37.00 to 48.95 | 0.05 | 0.1 |
|  | 15X | 35.84 to 47.39 | 0.28 | 0.09 |  | 40.03 to 59.33 | 0.1 | 0.41 |
|  | 16S | 33.39 to 45.75 | 0.03 | -0.05 |  | 35.65 to 51.49 | -0.21 | 0.21 |
|  | 16X | 35.66 to 46.63 | 0.54 | 0.22 |  | 39.34 to 53.96 | 0 | 0.01 |
| KW | 15S | 87.09 to 108.31 | 0.11 | 0.52 |  | 85.82 to 106.66 | 0.13 | -0.02 |
|  | 15X | 84.58 to 107.34 | 0.05 | 0.69 |  | 85.21 to 104.65 | 0.11 | 0.15 |
|  | 16S | 82.42 to 106.74 | -0.05 | 0.91 |  | 87.09 to 104.42 | 0.05 | -0.19 |
|  | 16X | 82.92 to 110.46 | 0.47 | 0.16 |  | 83.35 to 106.44 | 0.34 | 0 |
| KL | 15S | 105.53 to 130.16 | -0.28 | 0.52 |  | 97.27 to 122.43 | -0.24 | -0.17 |
|  | 15X | 114.60 to 143.34 | -0.73 | 1.33 |  | 106.19 to 134.03 | -0.18 | 0.76 |
|  | 16S | 105.26 to 132.92 | -0.79 | 0.68 |  | 107.66 to 132.34 | 0.06 | 0.13 |
|  | 16X | 102.70 to 135.00 | -0.13 | 0.08 |  | 104.07 to 131.63 | -0.26 | 0.43 |
| VW | 15S | 421.09 to 612.08 | 0.07 | 0.6 |  | 415.10 to 590.70 | 0.16 | 0.37 |
|  | 15X | 514.09 to 720.61 | 0.34 | 0.02 |  | 502.46 to 716.94 | 0.11 | 0.1 |
|  | 16S | 386.47 to 639.35 | 0.27 | 0.74 |  | 413.93 to 607.05 | 0.23 | 0.39 |
|  | 16X | 532.59 to 707.35 | 0.26 | 0.58 |  | 582.13 to 707.75 | 0.19 | -0.24 |
| HKW | 15S | 16.99 to 34.99 | 0.26 | 1.12 |  | 15.31 to 33.48 | 0.24 | 1.63 |
|  | 15X | 20.46 to 38.79 | 0.19 | 0.47 |  | 21.65 to 35.54 | 0.03 | -0.47 |
|  | 16S | 17.28 to 29.94 | -0.18 | -0.03 |  | 19.35 to 37.14 | 0.61 | 3.02 |
|  | 16X | 16.99 to 33.77 | 0.21 | 0.42 |  | 21.61 to 37.88 | -0.22 | 0.75 |
| YP | 15S | 799.56 to 1696.37 | -0.14 | 0.09 |  | 686.67 to 1402.38 | -0.37 | 0.52 |
|  | 15X | 816.25 to 2119.41 | -0.38 | 1.52 |  | 830.31 to 1797.25 | -0.12 | 0.52 |
|  | 16S | 426.83 to 1393.29 | -0.36 | 0.77 |  | 620.97 to 1489.04 | -0.21 | 0.15 |
|  | 16X | 806.88 to 1652.64 | 0.07 | -0.24 |  | 873.04 to 1694.46 | -0.35 | 0.36 |

^a^Trait refers to the name of each component of yield-related traits: RN, Row number; KN, Kernel number per row; KT, Kernel thickness; KW, Kernel width; KL, Kernel length; HKW, 100-kernel weight; VW, Volume weight; YP, Yield per plot

^b^Specific growing environments: 15S is 2015 Shijiazhuang; 15X is 2015 Xinxiang; 16S is 2016 Shijiazhuang; 16X is 2016 Xinxiang; C is joint analyses

**Supplementary Table S3** QTL detected for eight yield-related traits in RILs

| Trait Name^a^ | | Name^b^ | Env^c^ | Chr.^d^ | Marker interval^e^ | interval^f^ | LOD^g^ | | PVE^h^ | | ADD^i^ | CON^j^ | |
| --- | --- | --- | --- | --- | --- | --- | --- | --- | --- | --- | --- | --- | --- |
| RN | | *qRN1-1* | 16S | 1 | mk227-mk236 | 59.10-65.00 | | 4.17 | | 4.37 | 0.44 |  |  |
|  |  | *qRN1-4* | 15S/15X | 1 | mk530-mk578 | 221.15-237.25 | | 4.62 | | 5.38 | 0.43 |  |  |
|  |  | *qRN2-1* | 16X/C | 2 | mk872-mk890 | 7.70-10.85 | | 4.90 | | 5.52 | 0.41 | * |  |
|  |  | *qRN2-2* | 16S/16X/C | 2 | mk1053-mk1065 | 203.05-209.10 | | 5.07 | | 5.14 | -0.40 |  |  |
|  |  | *qRN3-2* | C | 3 | mk1599-mk1616 | 203.65-207.50 | | 4.06 | | 4.23 | 0.29 |  |  |
|  |  | *qRN5-2* | 15X/C | 5 | mk2296-mk2312 | 10.35-13.85 | | 5.21 | | 5.79 | 0.40 |  |  |
|  |  | *qRN5-4* | 15S/16S/16X | 5 | mk2543-mk2558 | 160.75-167.55 | | 4.99 | | 5.86 | 0.48 | * |  |
|  |  | *qRN8-1* | 15X | 8 | mk3626-mk3637 | 11.60-13.75 | | 3.95 | | 4.70 | 0.43 | * |  |
|  |  | *qRN9-1* | 15S/15X/16S/16X/C | 9 | mk3994-mk4043 | 7.85-16.85 | | 4.13 | | 4.71 | 0.37 | * |  |
|  |  | *qRN10-1* | 15S/15X | 10 | mk4367-mk4399 | 10.35-19.40 | | 4.72 | | 5.37 | -0.43 | * |  |
| KN | *qKN2* | 15X/16X/C | 2 | mk952-mk984 | 171.95-183.20 | | 4.00 | | 4.55 | -1.37 | * |  |  |
|  | *qKN3-2* | 15S | 3 | mk1477-mk1498 | 165.80-170.85 | | 3.24 | | 3.89 | -1.18 |  |  |  |
|  | *qKN4-2* | 16X | 4 | mk1908-mk1914 | 131.65-139.15 | | 4.15 | | 2.71 | 1.23 | * |  |  |
|  | *qKN5* | 15X/16S/16X/C | 5 | mk2553-mk2613 | 165.05-183.55 | | 4.30 | | 4.82 | 1.42 |  |  |  |
|  | *qKN9* | 15S | 9 | mk4131-mk4138 | 89.30-94.70 | | 4.45 | | 6.72 | 1.53 | * |  |  |
|  | *qKN10-1* | 16X | 10 | mk4372-mk4395 | 11.40-16.85 | | 4.34 | | 5.23 | 1.72 |  |  |  |
|  | *qKN10-2* | 15X/16S/C | 10 | mk4456-mk4465 | 91.25-102.60 | | 4.86 | | 6.02 | 1.60 | * |  |  |
| KT | *qKT3-3* | 15S/16S/C | 3 | mk1433-mk1495 | 152.70-170.25 | | 5.37 | | 5.43 | 1.74 |  |  |  |
|  | *qKT4-1* | 15S/16S | 4 | mk1837-mk1857 | 30.00-39.90 | | 4.66 | | 5.93 | -2.07 | * |  |  |
|  | *qKT4-2* | 15X/16X/C | 4 | mk1904-mk1910 | 128.15-134.4 | | 5.89 | | 6.76 | -1.92 |  |  |  |
|  | *qKT5-1* | 15S/16X | 5 | mk2555-mk2580 | 166.15-173.1 | | 4.55 | | 5.28 | -1.89 |  |  |  |
|  | *qKT6* | 15X/C | 6 | mk2778-mk2789 | 1.30-10.40 | | 3.84 | | 4.30 | -1.53 |  |  |  |
|  | *qKT7-1* | 15S/C | 7 | mk3339-mk3391 | 115.35-134.05 | | 3.52 | | 4.25 | 1.42 |  |  |  |
|  | *qKT10-1* | 16S | 10 | mk4360-mk4376 | 8.75-12.65 | | 5.93 | | 6.83 | -2.56 |  |  |  |
|  | *qKT10-2* | 15X | 10 | mk4464-mk4472 | 100.40-107.30 | | 3.63 | | 5.02 | -2.01 | * |  |  |
| KW | *qKW1-1* | 15X/16S/16X/C | 1 | mk102-mk170 | 19.25-35.35 | | 6.38 | | 6.89 | -2.28 | * |  |  |
|  | *qKW1-3* | 15S/16S | 1 | mk608-mk698 | 246.90-274.65 | | 3.35 | | 3.27 | -1.75 |  |  |  |
|  | *qKW3-2* | 15S | 3 | mk1327-mk1336 | 34.25-40.85 | | 6.13 | | 6.94 | 2.73 |  |  |  |
|  | *qKW3-3* | 15X/16S/16X/C | 3 | mk1443-mk1475 | 155.05-165.4 | | 8.54 | | 8.75 | 2.64 | * |  |  |
|  | *qKW4-3* | 16S/C | 4 | mk1989-mk2106 | 173.50-210.70 | | 3.66 | | 2.19 | 1.24 | * |  |  |
|  | *qKW4-4* | 15X | 4 | mk2160-mk2179 | 229.70-234.05 | | 3.98 | | 4.48 | 2.10 |  |  |  |
|  | *qKW6-1* | 16X | 6 | mk2778-mk2789 | 1.30-10.40 | | 3.18 | | 3.56 | -2.14 |  |  |  |
|  | *qKW6-3* | 15X | 6 | mk2829-mk2835 | 65.00-71.65 | | 3.91 | | 4.05 | -2.01 | * |  |  |
|  | *qKW6-4* | 15S | 6 | mk3039-mk3055 | 146.10-149.85 | | 5.15 | | 5.77 | -2.33 |  |  |  |
|  | *qKW7-1* | 16S | 7 | mk3314-mk3319 | 96.40-105.35 | | 6.21 | | 6.42 | 2.50 |  |  |  |
|  | *qKW7-2* | 15S/15X/16X/C | 7 | mk3370-mk3438 | 126.10-148.90 | | 6.23 | | 6.97 | 2.20 | * |  |  |
|  | *qKW8* | 15S/15X16S/16X/C | 8 | mk3862-mk3933 | 161.90-171.35 | | 5.44 | | 5.48 | -1.99 | * |  |  |
|  | *qKW10* | 16S/C | 10 | mk4464-mk4481 | 100.40-116.80 | | 5.48 | | 5.66 | -2.02 |  |  |  |
| KL | *qKL2* | C | 2 | mk832-mk849 | 1.50-3.65 | | 3.49 | | 3.20 | 1.48 | * |  |  |
|  | *qKL3-2* | 15S/C | 3 | mk1328-mk1346 | 34.80-51.95 | | 6.63 | | 6.05 | 2.63 |  |  |  |
|  | *qKL3-3* | 15X/16S/16X | 3 | mk1460-mk1477 | 159.05-165.8 | | 3.80 | | 5.94 | 3.14 |  |  |  |
|  | *qKL3-4* | 15S/16S/C | 3 | mk1626-mk1673 | 209.10-217.25 | | 7.53 | | 7.84 | 2.72 | * |  |  |
|  | *qKL4-1* | 15X | 4 | mk1837-mk1847 | 30.00-34.95 | | 4.40 | | 5.44 | 2.86 | * |  |  |
|  | *qKL4-2* | 16X | 4 | mk1893-mk1896 | 91.60-101.10 | | 3.27 | | 3.00 | 2.45 |  |  |  |
|  | *qKL4-3* | 15S/C | 4 | mk1936-mk1958 | 152.25-159.20 | | 4.44 | | 5.19 | 2.25 | * |  |  |
|  | *qKL4-4* | 16S | 4 | mk2170-mk2196 | 231.75-236.90 | | 3.12 | | 4.11 | 2.35 |  |  |  |
|  | *qKL5-2* | 15S | 5 | mk2640-mk2684 | 190.85-201.6 | | 3.03 | | 3.30 | -2.08 |  |  |  |
|  | *qKL7-1* | 16S | 7 | mk3249-mk3260 | 24.50-29.80 | | 3.23 | | 4.25 | 2.38 |  |  |  |
|  | *qKL7-2* | 15S/15X/16X/C | 7 | mk3397-mk3449 | 137.05-151.50 | | 5.78 | | 6.19 | 2.53 | * |  |  |
|  | *qKL7-4* | 15S | 7 | mk3514-mk3531 | 163.95-167.15 | | 4.15 | | 4.54 | 2.52 |  |  |  |
|  | *qKL10-2* | 15X/C | 10 | mk4438-mk4486 | 82.55-118.65 | | 3.46 | | 3.59 | -2.01 | * |  |  |
| VW | *qVW1* | 16X | 1 | mk568-mk583 | 234.05-239.30 | | 4.25 | | 2.57 | -11.82 |  |  |  |
|  | *qVW3-2* | 16S | 3 | mk1423-mk1438 | 148.65-154.10 | | 3.22 | | 5.06 | -20.44 |  |  |  |
|  | *qVW4-2* | 15S | 4 | mk1955-mk1964 | 157.40-163.40 | | 3.45 | | 4.22 | -19.03 |  |  |  |
|  | *qVW6-2* | 15S | 6 | mk2854-mk2870 | 83.55-89.85 | | 3.14 | | 3.53 | 17.57 |  |  |  |
|  | *qVW8-1* | 15X | 8 | mk3673-mk3679 | 74.30-79.65 | | 4.08 | | 4.65 | -21.86 |  |  |  |
|  | *qVW8-2* | 15S | 8 | mk3927-mk3947 | 170.75-173.05 | | 3.45 | | 4.06 | 18.59 |  |  |  |
|  | *qVW10-1* | 15X/16S/C | 10 | mk4332-mk4379 | 4.05-34.60 | | 5.07 | | 4.26 | 13.77 |  |  |  |
|  | *qVW10-2* | 15S | 10 | mk4456-mk4461 | 91.25-96.90 | | 5.22 | | 7.85 | 26.58 |  |  |  |
|  | *qVW10-3* | 16X | 10 | mk4524-mk4545 | 136.15-140.55 | | 3.42 | | 3.41 | 13.63 |  |  |  |
| HKW | *qHKW1-1* | 16X/C | 1 | mk105-mk139 | 19.85-26.85 | | 6.02 | | 5.84 | -1.43 | * |  |  |
|  | *qHKW1-2* | 15X/16S | 1 | mk287-mk298 | 86.30-93.15 | | 3.49 | | 4.04 | -1.45 |  |  |  |
|  | *qHKW1-3* | 15S/16S/16X/C | 1 | mk612-mk638 | 249.15-277.15 | | 4.73 | | 4.51 | -1.14 | * |  |  |
|  | *qHKW2-1* | C | 2 | mk864-mk882 | 6.05-9.65 | | 4.19 | | 4.68 | -0.94 | * |  |  |
|  | *qHKW2-3* | 16X | 2 | mk1084-mk1108 | 213.10-218.20 | | 4.33 | | 4.20 | -1.46 |  |  |  |
|  | *qHKW3-2* | 15S/15X/16S/16X/C | 3 | mk1449-mk1483 | 156.45-167.60 | | 8.66 | | 8.89 | 1.64 |  |  |  |
|  | *qHKW5-2* | 15X/15S/C | 5 | mk2552-mk2619 | 164.95-184.45 | | 3.76 | | 4.36 | -1.21 |  |  |  |
|  | *qHKW6-1* | 15X | 6 | mk2829-mk2835 | 65.00-71.65 | | 3.26 | | 3.98 | -1.61 |  |  |  |
|  | *qHKW7-1* | 16S | 7 | mk3248-mk3260 | 24.15-29.80 | | 4.76 | | 5.04 | 1.44 |  |  |  |
|  | *qHKW7-3* | 15S/16X/C | 7 | mk3401-mk3461 | 138.55-153.95 | | 7.97 | | 8.01 | 1.56 | * |  |  |
|  | *qHKW8* | 16X/C | 8 | mk3859-mk3892 | 161.35-166.15 | | 4.38 | | 4.14 | -1.12 | * |  |  |
|  | *qHKW10* | 15X/16S/C | 10 | mk4435-mk4488 | 82.00-118.90 | | 4.26 | | 4.76 | -1.44 |  |  |  |
| YP | *qYP1* | 15S/C | 1 | mk466-mk488 | 201.95-207.95 | | 3.54 | | 4.04 | -37.90 |  |  |  |
|  | *qYP2-1* | 15X | 2 | mk952-mk965 | 171.95-177.90 | | 3.21 | | 3.94 | -55.96 |  |  |  |
|  | *qYP2-3* | 16S/16X/C | 2 | mk1013-mk1044 | 190.40-199.05 | | 4.64 | | 5.73 | -50.69 |  |  |  |
|  | *qYP3-1* | 16X | 3 | mk1570-mk1585 | 194.75-200.20 | | 4.84 | | 6.74 | 77.77 |  |  |  |
|  | *qYP4* | 15S | 4 | mk1731-mk1751 | 0.35-3.75 | | 3.32 | | 3.51 | -41.74 |  |  |  |
|  | *qYP5-1* | 15X | 5 | mk2595-mk2609 | 176.30-182.30 | | 3.62 | | 4.60 | 60.42 |  |  |  |
|  | *qYP6-1* | 15X | 6 | mk2859-mk2870 | 85.55-89.85 | | 6.13 | | 8.52 | -97.00 |  |  |  |
|  | *qYP6-2* | 15X | 6 | mk2914-mk2929 | 104.35-108.90 | | 3.67 | | 4.91 | 72.54 |  |  |  |
|  | *qYP7* | 15S | 7 | mk3475-mk3495 | 157.05-160.75 | | 5.70 | | 6.96 | 59.03 |  |  |  |
|  | *qYP9-1* | 16S/C | 9 | mk4144-mk4167 | 98.10-105.55 | | 3.92 | | 5.13 | 43.46 |  |  |  |

^a^Trait refers to the name of each component of yield-related traits: RN, Row number; KN, Kernel number per row; KT, Kernel thickness; KW, Kernel width; KL, Kernel length; HKW, 100-kernel weight; VW, Volume weight; YP, Yield per plot

^b^The name of each QTL is a composite of the influenced trait: RN, KN, KT, KW, KL, HKW, VW or YP

^c^Chr., chromosome

^d^Specific growing environment: 15S is 2015 Shijiazhuang; 15X is 2015 Xinxiang; 16S is 2016 Shijiazhuang; 16X is 2016 Xinxiang; C is joint analyses

^e^Marker interval, the markers flanking the QTL

^f^Interval, confidence interval between two bin markers

^g^LOD, the logarithm of odds score

^h^ PVE, the phenotypic variance explained by an individual QTL

^i^ADD, the value of additive effects. LOD scores, PVE values, and ADD values are shown as mean values for QTL with multiple effects

^j^CON， QTL consistent with previous study represented by the symbol *.

**Supplementary Table S4** QTL detected for the GCA effects of eight yield-related traits

| Trait Name^a^ | Name^b^ | Env^c^ | Chr.^d^ | Marker interval^e^ | interval^f^ | LOD^g^ | PVE^h^ | ADD^i^ |
| --- | --- | --- | --- | --- | --- | --- | --- | --- |
| RN | *qRN1-2* | 16X | 1 | mk249-mk264 | 70.85-76.70 | 3.45 | 3.85 | -0.01 |
|  | *qRN1-3* | 16X | 1 | mk430-mk444 | 190.95-196.50 | 3.75 | 3.49 | 0.00 |
|  | *qRN2-1* | 15X/C | 2 | mk876-mk904 | 8.65-13.30 | 4.34 | 3.46 | 0.08 |
|  | *qRN3-1* | C | 3 | mk1298-mk1313 | 21.45-25.65 | 8.12 | 7.03 | -0.21 |
|  | *qRN3-3* | 16S | 3 | mk1663-mk1681 | 215.40-220.15 | 3.78 | 4.72 | -0.01 |
|  | *qRN4-1* | C | 4 | mk1749-mk1784 | 3.45-11.90 | 3.53 | 3.68 | 0.15 |
|  | *qRN4-2* | 15S | 4 | mk1976-mk1993 | 170.75-175.15 | 4.03 | 3.07 | 0.00 |
|  | *qRN4-3* | C | 4 | mk2047-mk2117 | 189.65-216.40 | 5.47 | 3.71 | -0.15 |
|  | *qRN5-1* | 15X | 5 | mk2246-mk2253 | 3.05-4.15 | 5.05 | 4.93 | -0.01 |
|  | *qRN5-2* | 16S/C | 5 | mk2286-mk2367 | 8.75-26.20 | 3.35 | 3.46 | 0.06 |
|  | *qRN5-3* | 16S | 5 | mk2493-mk2496 | 105.15-119.70 | 3.86 | 4.03 | -0.01 |
|  | *qRN5-4* | C | 5 | mk2519-mk2596 | 147.05-176.95 | 3.71 | 1.10 | 0.09 |
|  | *qRN6* | C | 6 | mk3078-mk3095 | 156.15-159.15 | 8.53 | 7.89 | 0.22 |
|  | *qRN7* | C | 7 | mk3154-mk3169 | 0.35-2.65 | 4.22 | 3.02 | -0.14 |
|  | *qRN8-2* | C | 8 | mk3892-mk3916 | 166.15-169.15 | 4.36 | 2.84 | -0.13 |
|  | *qRN9-1* | C | 9 | mk4010-mk4039 | 10.90-16.00 | 7.58 | 5.83 | 0.19 |
|  | *qRN9-2* | 16X | 9 | mk4255-mk4271 | 133.50-136.75 | 4.97 | 3.19 | 0.00 |
|  | *qRN10-2* | C | 10 | mk4458-mk4464 | 95.05-100.40 | 10.20 | 8.51 | -0.23 |
| KN | *qKN1-1* | C | 1 | mk64-mk91 | 12.55-17.55 | 3.15 | 3.81 | -0.38 |
|  | *qKN1-2* | 15X | 1 | mk146-mk165 | 28.30-33.75 | 4.73 | 5.41 | -0.15 |
|  | *qKN1-3* | 15S | 1 | mk444-mk459 | 196.50-200.15 | 4.97 | 4.90 | -0.13 |
|  | *qKN1-4* | C | 1 | mk484-mk505 | 207.05-213.05 | 4.75 | 4.80 | -0.43 |
|  | *qKN1-5* | 16X | 1 | mk582-mk597 | 238.45-244.00 | 3.49 | 4.10 | 0.12 |
|  | *qKN1-6* | C | 1 | mk686-mk700 | 270.35-275.00 | 9.06 | 8.43 | 0.57 |
|  | *qKN3-1* | 15X/C | 3 | mk1239-mk1302 | 9.95-22.40 | 4.86 | 4.51 | -0.25 |
|  | *qKN3-3* | C | 3 | mk1534-mk1555 | 183.35-188.50 | 4.94 | 3.48 | -0.36 |
|  | *qKN3-4* | 16S | 3 | mk1659-mk1681 | 214.70-220.15 | 3.20 | 3.71 | -0.17 |
|  | *qKN4-1* | 15X | 4 | mk1873-mk1876 | 64.55-70.55 | 3.37 | 3.76 | 0.12 |
|  | *qKN6* | 15X | 6 | mk3116-mk3132 | 162.10-164.90 | 3.48 | 3.13 | 0.11 |
|  | *qKN7-1* | C | 7 | mk3337-mk3344 | 113.10-117.70 | 11.85 | 12.24 | -0.67 |
|  | *qKN7-2* | 16S | 7 | mk3388-mk3399 | 132.60-138.35 | 3.70 | 3.63 | -0.16 |
|  | *qKN7-3* | C | 7 | mk3581-mk3593 | 174.45-176.65 | 3.72 | 2.73 | 0.32 |
|  | *qKN8-1* | 16S | 8 | mk3787-mk3792 | 131.45-137.40 | 9.33 | 9.10 | 0.26 |
|  | *qKN8-2* | 16S/C | 8 | mk3925-mk3938 | 170.45-171.95 | 4.52 | 4.05 | 0.24 |
| KT | *qKT1-1* | 16S | 1 | mk352-mk364 | 160.40-166.70 | 3.18 | 1.63 | -0.03 |
|  | *qKT1-2* | 15S/16X | 1 | mk472-mk493 | 203.55-208.95 | 4.89 | 5.72 | 0.01 |
|  | *qKT1-3* | C | 1 | mk677-mk697 | 266.35-274.40 | 5.54 | 4.12 | -0.53 |
|  | *qKT2* | C | 2 | mk837-mk847 | 2.05-3.20 | 6.80 | 7.25 | -0.71 |
|  | *qKT3-1* | C | 3 | mk1201-mk1212 | 2.45-4.80 | 3.02 | 2.00 | 0.39 |
|  | *qKT3-2* | 16S | 3 | mk1423-mk1438 | 148.65-154.10 | 3.37 | 3.61 | 0.05 |
|  | *qKT3-3* | C | 3 | mk1442-mk1463 | 154.85-160.25 | 6.08 | 2.94 | 0.47 |
|  | *qKT4-2* | 15X/C | 4 | mk1903-mk1922 | 126.50-144.75 | 8.54 | 7.63 | -0.45 |
|  | *qKT5-2* | 15X/C | 5 | mk2696-mk2728 | 203.70-210.80 | 3.64 | 2.47 | 0.21 |
|  | *qKT7-1* | C | 7 | mk3337-mk3348 | 113.10-118.90 | 5.22 | 5.50 | 0.63 |
|  | *qKT7-2* | 15S | 7 | mk3538-mk3560 | 168.25-171.75 | 3.23 | 4.53 | -0.04 |
|  | *qKT8* | C | 8 | mk3805-mk3848 | 147.05-159.75 | 3.81 | 2.17 | 0.39 |
|  | *qKT9* | C | 9 | mk4040-mk4051 | 16.20-18.45 | 3.83 | 2.76 | -0.43 |
|  | *qKT10-1* | C | 10 | mk4397-mk4402 | 18.10-23.60 | 5.37 | 4.56 | -0.56 |
| KW | *qKW1-2* | 15X | 1 | mk286-mk297 | 85.30-90.75 | 4.98 | 2.41 | -0.03 |
|  | *qKW1-3* | C | 1 | mk665-mk681 | 262.90-268.25 | 5.45 | 5.56 | -0.92 |
|  | *qKW3-1* | C | 3 | mk1282-mk1317 | 18.85-27.80 | 4.22 | 0.28 | 0.29 |
|  | *qKW3-2* | C | 3 | mk1331-mk1343 | 36.35-49.85 | 3.55 | 0.01 | 0.07 |
|  | *qKW3-3* | C | 3 | mk1426-mk1454 | 149.65-157.55 | 5.25 | 1.37 | 0.59 |
|  | *qKW3-4* | 16X | 3 | mk1605-mk1608 | 205.10-205.80 | 5.41 | 3.20 | 0.05 |
|  | *qKW4-1* | C | 4 | mk1832-mk1860 | 26.30-43.90 | 4.06 | 0.35 | 0.41 |
|  | *qKW4-2* | C | 4 | mk1870-mk1907 | 58.15-130.70 | 3.41 | 0.13 | 0.25 |
|  | *qKW4-3* | C | 4 | mk2065-mk2084 | 195.20-201.10 | 8.23 | 4.78 | 0.86 |
|  | *qKW5-1* | C | 5 | mk2434-mk2486 | 63.80-90.65 | 3.10 | 0.06 | -0.16 |
|  | *qKW5-2* | C | 5 | mk2496-mk2532 | 119.70-154.8 | 3.83 | 0.64 | -0.54 |
|  | *qKW6-2* | 16S | 6 | mk2809-mk2819 | 32.80-38.85 | 3.16 | 3.34 | -0.04 |
|  | *qKW6-4* | 15X/C | 6 | mk3034-mk3053 | 140.70-149.65 | 9.06 | 8.69 | -0.65 |
|  | *qKW6-5* | 16X/C | 6 | mk3131-mk3153 | 164.75-168.75 | 4.71 | 4.31 | -0.46 |
|  | *qKW7-2* | C | 7 | mk3374-mk3385 | 127.05-131.10 | 7.89 | 6.32 | 0.98 |
|  | *qKW7-3* | 15S | 7 | mk3419-mk3436 | 143.30-148.55 | 3.35 | 2.31 | 0.03 |
|  | *qKW7-4* | 16S | 7 | mk3523-mk3545 | 165.65-168.95 | 4.18 | 3.21 | -0.04 |
|  | *qKW9* | 15X/C | 9 | mk3969-mk4040 | 2.70-16.20 | 3.51 | 3.24 | -0.31 |
| KL | *qKL1-1* | C | 1 | mk290-mk336 | 88.20-148.65 | 3.09 | 2.42 | -0.67 |
|  | *qKL1-2* | 16X/C | 1 | mk433-mk468 | 191.75-202.6 | 6.13 | 4.44 | -0.53 |
|  | *qKL1-3* | 15X | 1 | mk573-mk586 | 234.85-240.5 | 4.01 | 3.82 | -0.11 |
|  | *qKL1-4* | 16S | 1 | mk701-mk711 | 275.20-277.85 | 3.94 | 0.08 | -0.02 |
|  | *qKL2* | 15S/C | 2 | mk837-mk884 | 2.05-10.05 | 7.67 | 7.01 | 0.74 |
|  | *qKL3-1* | 15S | 3 | mk1293-mk1314 | 20.80-26.20 | 3.55 | 3.81 | 0.16 |
|  | *qKL3-4* | 16X/C | 3 | mk1600-mk1724 | 204.25-230.45 | 4.12 | 4.07 | 0.47 |
|  | *qKL4-1* | C | 4 | mk1838-mk1850 | 30.25-36.20 | 5.29 | 5.91 | 1.00 |
|  | *qKL4-4* | 15S | 4 | mk2146-mk2165 | 225.5-230.85 | 3.16 | 3.51 | 0.15 |
|  | *qKL5-1* | 15S | 5 | mk2588-mk2605 | 174.45-180.00 | 3.28 | 3.63 | -0.15 |
|  | *qKL5-2* | C | 5 | mk2700-mk2715 | 204.35-206.65 | 4.95 | 3.59 | -0.77 |
|  | *qKL6* | 15X | 6 | mk2776-mk2787 | 0.35-4.10 | 3.16 | 4.22 | -0.12 |
|  | *qKL7-3* | 16S | 7 | mk3470-mk3496 | 155.90-160.90 | 3.82 | 2.04 | -0.09 |
|  | *qKL8-1* | 15S | 8 | mk3792-mk3799 | 137.40-143.55 | 4.80 | 5.27 | 0.18 |
|  | *qKL8-2* | 16S | 8 | mk3869-mk3899 | 163.45-166.95 | 3.75 | 3.26 | -0.12 |
|  | *qKL9* | C | 9 | mk4169-mk4259 | 106.10-134.25 | 3.91 | 2.60 | 0.66 |
|  | *qKL10-1* | 15X | 10 | mk4328-mk4337 | 3.05-4.65 | 3.49 | 3.52 | -0.11 |
|  | *qKL10-2* | C | 10 | mk4456-mk4462 | 91.25-97.7 | 9.89 | 9.50 | -1.27 |
| VW | *qVW3-1* | 15S | 3 | mk1321-mk1332 | 31.65-37.05 | 5.83 | 7.45 | -1.26 |
|  | *qVW3-3* | 15X/16X | 3 | mk1465-mk1499 | 161.00-171.25 | 3.45 | 1.76 | 0.75 |
|  | *qVW3-4* | 16S | 3 | mk1566-mk1579 | 193.00-198.40 | 3.02 | 3.37 | 1.13 |
|  | *qVW4-1* | C | 4 | mk1839-mk1851 | 31.00-36.70 | 6.94 | 6.73 | -5.51 |
|  | *qVW4-3* | 16S | 4 | mk2031-mk2034 | 183.35-185.55 | 5.63 | 4.26 | -1.28 |
|  | *qVW5-1* | 16X | 5 | mk2439-mk2454 | 67.20-73.70 | 3.24 | 0.04 | -0.09 |
|  | *qVW5-2* | C | 5 | mk2625-mk2775 | 187.20-217.35 | 3.14 | 2.62 | -3.42 |
|  | *qVW6-1* | 15X/C | 6 | mk2776-mk2787 | 0.35-4.10 | 4.89 | 5.58 | 3.38 |
|  | *qVW10-1* | 16S | 10 | mk4399-mk4404 | 19.40-25.75 | 7.28 | 7.96 | 1.75 |
|  | *qVW10-2* | 15S/C | 10 | mk4464-mk4473 | 100.40-108.35 | 8.92 | 9.31 | 4.44 |
| HKW | *qHKW1-3* | 16X/C | 1 | mk665-mk687 | 262.90-271.20 | 10.37 | 10.78 | -0.46 |
|  | *qHKW2-2* | 16S | 2 | mk1012-mk1024 | 190.25-193.25 | 4.76 | 4.70 | 0.11 |
|  | *qHKW3-1* | C | 3 | mk1274-mk1302 | 16.90-22.40 | 8.14 | 2.74 | 0.37 |
|  | *qHKW3-2* | 15X/C | 3 | mk1416-mk1499 | 142.55-171.25 | 4.31 | 5.57 | 0.26 |
|  | *qHKW3-3* | 15S | 3 | mk1670-mk1676 | 216.80-217.65 | 5.65 | 3.22 | 0.10 |
|  | *qHKW4* | 15X/C | 4 | mk1736-mk1763 | 1.55-6.10 | 3.66 | 4.34 | -0.26 |
|  | *qHKW5-1* | 16S/16X | 5 | mk2257-mk2354 | 4.95-21.60 | 6.51 | 5.12 | 0.12 |
|  | *qHKW6-1* | 16S | 6 | mk2829-mk2835 | 65.00-71.65 | 3.69 | 4.32 | -0.10 |
|  | *qHKW6-2* | C | 6 | mk3075-mk3153 | 155.15-168.75 | 4.61 | 3.27 | -0.35 |
|  | *qHKW7-2* | C | 7 | mk3370-mk3386 | 126.1-131.55 | 8.89 | 7.49 | 0.53 |
|  | *qHKW9* | C | 9 | mk4005-mk4045 | 9.55-17.25 | 3.25 | 2.51 | -0.31 |
| YP | *qYP1* | 15S/C | 1 | mk444-mk464 | 196.50-201.10 | 7.47 | 7.38 | -12.41 |
|  | *qYP2-2* | 15S | 2 | mk980-mk998 | 181.65-186.40 | 3.16 | 4.17 | 5.46 |
|  | *qYP3-2* | 15S | 3 | mk1619-mk1643 | 208.05-212.20 | 3.64 | 4.29 | 5.55 |
|  | *qYP3-3* | 16S | 3 | mk1665-mk1676 | 216.05-217.65 | 3.71 | 3.46 | -4.96 |
|  | *qYP4* | 15X | 4 | mk1731-mk1740 | 0.35-2.20 | 4.88 | 5.86 | -6.66 |
|  | *qYP5-2* | 15S/16X | 5 | mk2724-mk2755 | 209.40-214.45 | 3.84 | 3.54 | -4.96 |
|  | *qYP6-3* | 16S | 6 | mk2954-mk2973 | 115.75-121.05 | 3.17 | 3.82 | -5.17 |
|  | *qYP8-1* | 16X | 8 | mk3661-mk3663 | 51.50-59.50 | 6.05 | 5.33 | -6.17 |
|  | *qYP8-2* | 16S | 8 | mk3787-mk3792 | 131.45-137.40 | 5.02 | 6.09 | 6.56 |
|  | *qYP9-2* | C | 9 | mk4288-mk4294 | 149.15-153.55 | 3.83 | 5.23 | 13.86 |
|  | *qYP10* | C | 10 | mk4432-mk4449 | 80.35-85.70 | 7.30 | 9.18 | -18.62 |

^a^Trait refers to the name of each component of yield-related traits: RN, Row number; KN, Kernel number per row; KT, Kernel thickness; KW, Kernel width; KL, Kernel length; HKW, 100-kernel weight; VW, Volume weight; YP, Yield per plot

^b^The name of each QTL is a composite of the influenced trait: RN, KN, KT, KW, KL, HKW, VW or YP

^c^Chr., chromosome

^d^Specific growing environment: 15S is 2015 Shijiazhuang; 15X is 2015 Xinxiang; 16S is 2016 Shijiazhuang; 16X is 2016 Xinxiang; C representing joint analyses

^e^Marker interval, the markers flanking the QTL

^f^Interval, confidence interval between two bin markers

^g^LOD, the logarithm of odds score

^h^ PVE, the phenotypic variance explained by an individual QTL

^i^ADD, the value of additive effects. LOD scores, PVE values, and ADD values are shown as mean values for QTL with multiple effects


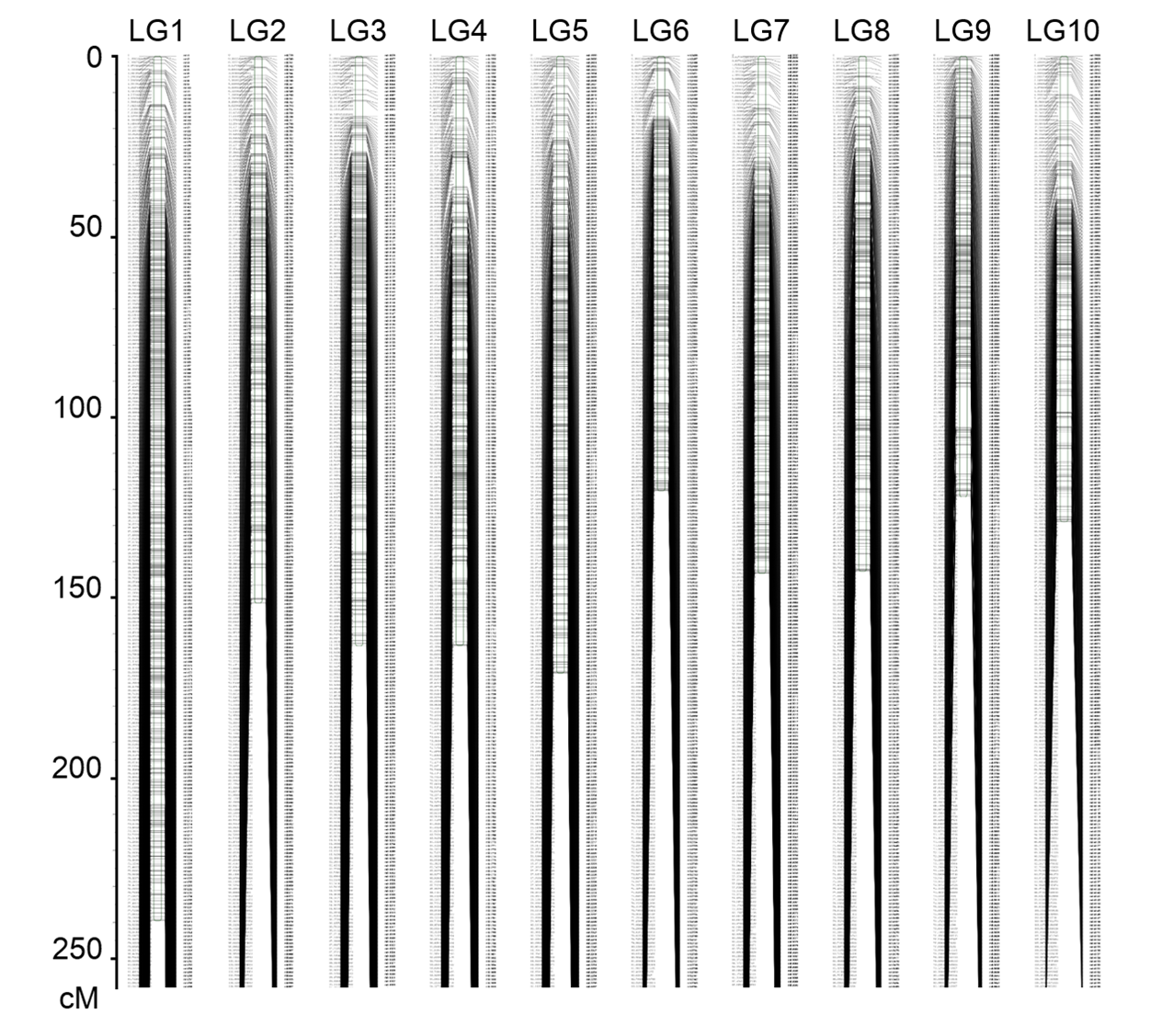


Figure S1 The high density genetic map created derived from 4602 bin markers (Zhou *et al.*, 2016)
